# Macrophage elastase (MMP12) critically contributes to the development of subretinal fibrosis

**DOI:** 10.1186/s12974-022-02433-x

**Published:** 2022-04-05

**Authors:** Caijiao Yi, Jian Liu, Wen Deng, Chang Luo, Jinyan Qi, Mei Chen, Heping Xu

**Affiliations:** 1 Aier School of Ophthalmology, Central South University, Changsha, 410000 China; 2 Aier Institute of Optometry and Vision Science, Changsha, 410000 China; 3 The Wellcome-Wolfson Institute for Experimental Medicine, School of Medicine, Dentistry and Biomedical Sciences, Queen’s University Belfast, 97 Lisburn Road, Belfast, BT9 7BL UK

**Keywords:** Age-related macular degeneration, Macular fibrosis, Inflammation, Matrix metalloproteinase-12, RNA sequencing

## Abstract

**Background:**

Macular subretinal fibrosis is the end-stage complication of neovascular age-related macular degeneration (nAMD). We previously developed a mouse model of two-stage laser-induced subretinal fibrosis that mimics closely the dynamic course of macular fibrosis in nAMD patients. This study was aimed to understand the molecular mechanism of subretinal fibrosis.

**Methods:**

Subretinal fibrosis was induced in C57BL/6J mice using the two-stage laser-induced protocol. Twenty days later, eyes were collected and processed for RNA sequencing (RNA-seq) analysis. DESeq2 was used to determine the differentially expressed genes (DEGs). Gene Ontology (GO) and KEGG were used to analyze the enriched pathways. The expression of the selected DEGs including *Mmp12* was verified by qPCR. The expression of MMP12 in subretinal fibrosis of mouse and nAMD donor eyes was examined by immunofluorescence and confocal microscopy. The expression of collagen 1, αSMA and fibronectin and cytokines in bone marrow-derived macrophages from control and subretinal fibrosis mice were examined by qPCR, immunocytochemistry and Luminex multiplex cytokine assay. The MMP12 specific inhibitor MMP408 was used to evaluate the effect of MMP12 on TGFβ-induced macrophage-to-myofibroblast transition (MMT) in vitro and its role in subretinal fibrosis in vivo.

**Results:**

RNA-seq analysis of RPE-choroid from subretinal fibrosis eyes uncovered 139 DEGs (fold change log2(fc) ≥ 0.5, FDR < 0.05), including 104 up-regulated and 35 were down-regulated genes. The top 25 enrichment GO terms were related to inflammation, blood vessels/cardiovascular development and angiogenesis. One of the most significantly upregulated genes, *Mmp12*, contributed to 12 of the top 25 GO terms. Higher levels of MMP12 were detected in subretinal fibrotic lesions in nAMD patients and the mouse model, including in F4/80^+^ or Iba1^+^ macrophages. BMDMs from subretinal fibrosis mice expressed higher levels of MMP12, collagen-1, αSMA and fibronectin. MMP408 dose-dependently suppressed TGFβ-induced MMT in BMDMs. In vivo treatment with MMP408 (5 mg/kg) significantly reduced subretinal fibrosis accompanied by reduced F4/80^+^ macrophage infiltration.

**Conclusions:**

MMP12 critically contributes to the development of subretinal fibrosis, partially through promoting MMT.

**Supplementary Information:**

The online version contains supplementary material available at 10.1186/s12974-022-02433-x.

## Introduction

Macular subretinal fibrosis is the most severe form of end-stage complication of neovascular age-related macular degeneration (nAMD), a leading cause of blindness in the elderly in developed countries [1, 2]. Without any therapy, all nAMD patients eventually developed macular fibrosis [3]. With current anti-VEGF therapy, between 20–60% of nAMD patients develop subretinal fibrosis in 2–7 years [4–7]. Unlike the fibrotic scar that heals a skin wound, subretinal fibrosis is a vascularized lesion with a significant number of immune cells and myofibroblasts and excessive extracellular matrix protein deposition [8, 9], a typical “hot fibrosis” [10]. The “hot fibrosis” has a dynamic and active myofibroblast-macrophage (immune cell) interaction and this continued inflammatory response damages remaining healthy cells such as retinal pigment epithelial (RPE) cells and photoreceptors leading to irreversible sight loss. The molecular mechanism leading to the infiltration, activation and accumulation of myofibroblasts remains poorly defined. Because of this, no medication is currently available to prevent or treat macular fibrosis secondary to nAMD.

Clinical studies have shown that a large lesion size, poor baseline vision, higher rates of lesion activity (e.g., macular oedema, haemorrhage) [7, 11, 12], and longer interval between diagnosis and treatment [4] are risk factors of macular fibrosis in nAMD. We and others have reported that macular fibrosis in nAMD is associated with higher plasma levels of lipocalin 2 [13], complement fragments C3a, C4a and C5a [14] and lower serum levels of 25-hydroxyvitamin D [15]. Vitamin D has an important role in regulating immune cell functions [16]. We also found that the percentage of circulating CD4^+^ T cells in nAMD patients without macular fibrosis was lower compared to that in patients with subretinal fibrosis [17]. These clinical observations suggest a link between dysregulated or uncontrolled inflammation and macular fibrosis in nAMD.

We hypothesized that the higher rates of lesion activity (e.g., multiple episodes of macular oedema and haemorrhages) in nAMD may lead to prolonged chronic inflammation. The sustained inflammatory response plays an important role in converting the neovascular membrane into a fibrovascular lesion and the development of macular fibrosis [9, 18]. Based on this hypothesis, we developed a mouse model of two-stage laser-induced subretinal fibrosis [19]. In this model, choroidal neovascularization (CNV) was induced by the first laser burn of the Bruch’s membrane. 7–10 days later (when CNV reached peak size), a second laser burn was introduced to the CNV. The second laser burn induces leakage and haemorrhage and mimics the higher rates of disease activity in nAMD patients with macular fibrosis. This protocol resulted in the development of subretinal fibrovascular membranes that lasted for more than 40 days [19]. We believe this model mirrors closely the dynamic course of macular fibrosis in nAMD patients and is an ideal tool to study disease pathogenesis.

The current study was aimed to understand the molecular pathways involved in the development of subretinal fibrosis in our two-stage laser model. Using RNA sequencing (RNA-seq) technology, we identified matrix metalloproteinase 12 (*Mmp12*), also known as macrophage elastase, as one of the most significantly upregulated genes in subretinal fibrosis. Further in vitro and in vivo studies showed that MMP12 was involved in TGFβ-induced macrophage-to-myofibroblast transition (MMT) and blocking MMP12 significantly reduced subretinal fibrosis.

## Materials and methods

### Animals

C57BL/6J male mice aged between 8 and 10 weeks were provided by SJA Laboratory Animal Co., Ltd (Changsha, China) and raised in specific pathogen-free conditions on a 12-h day/night cycle with free access to food and water in the Department of Laboratory Animals of Central South University, China. All animal-related procedures were conducted following the Association for Research in Vision and Ophthalmology (ARVO) Statement for the Use of Animals in Ophthalmic and Vision Research and the protocols were approved by the Animal Welfare Ethics Committee of Central South University.

### Induction of subretinal fibrosis

Subretinal fibrosis was induced in mice using the two-stage laser protocol described previously by us with slight modifications [19]. Briefly, mice were anaesthetized with an intraperitoneal injection of sodium pentobarbital (60 mg/kg, Sigma Aldrich, St. Louis, MO), and pupils were dilated with the eyedrop containing 0.5% tropicamide and 0.5% phenylephrine (Santen Pharmaceutical Co., Ltd., Osaka, Japan). Carboxymethylcellulose sodium (Allergan Pharmaceutical Co., Ltd., County Mayo, Ireland) was used to moisten the ocular surface. Laser-induced choroidal neovascularization (CNV) was conducted using a photocoagulator (Topcon, Tokyo, Japan) (200 mv power, 100 ms duration and 60 μm spot size). Four laser burns were conducted around the optic head (at least two optic disc diameters away from the optic nerve head) in each eye. 10 days later, a second laser burn was applied to each lesion.

### RNA extraction, library preparation, and sequencing

Twenty days after the second laser, total RNAs were extracted from RPE-choroid using miRNeasy Micro Kit (Cat: 1071023, Qiagen, Dusseldorf, Germany) according to the manufacturer’s instructions. The RNA purity was determined by NanoDrop 2000 spectrophotometer (Thermo Fisher Scientific, Waltham, MA), RNA quantity was measured using Qubit2.0 Fluorometer (Invitrogen, Carlsbad, CA) and the RNA integrity was determined by Agilent 2100 bioanalyzer (Agilent Technologies, Palo Alto, CA). The cDNA libraries from 3 subretinal fibrosis mice and 3 healthy control mice were sequenced using the Illumina sequencing platform by Genedenovo Biotechnology Co., Ltd (Guangzhou, China). Low-quality reads or reads containing adapter and ploy-N were filtered by using the FASTP (Version 0.18.0, Haplox, Shenzhen, China). StringTie was used to count the numbers of reads mapped to each gene. Gene expression was calculated and expressed as fragments per kilobase of transcript per million mapped reads (FPKM). DESeq2 was used to determine the differentially expressed genes (DEGs) using the criteria of false discovery rate (FDR) < 0.05 (the *p*-value adjusted by the Benjamini–Hochberg method), log2(fc) ≥ 0.5, and count > 50 in at least 2 samples.

### Bioinformatics analysis

The free R language software (Version 3.6.3, https://www.r-project.org) was used to create the volcano plot of DEGs and the most significantly up- or down-regulated genes were labelled. Gene Ontology (GO) function analysis and Kyoto Encyclopedia of Genes and Genomes (KEGG) pathway analysis of DEGs were performed using the free online platform OmicShare (https://www.omicshare.com/tools). GO terms with FDR < 0.05 and KEGG pathways with *p* < 0.05 were considered significantly enriched. A protein–protein interaction (PPI) network of DEGs was searched in the STRING database with a confidence score > 0.4. The top 30 genes with the highest connection degree were calculated by the Cytohubba plugin and visualized in Cytoscape (Version3.8.0, Oracle, Redwood City, CA).

### Quantitative real-time PCR verification

Total RNA was extracted from RPE/choroid tissue as described above. In addition, we also extracted total RNA from bone marrow-derived macrophages (BMDMs) using the total RNA Kit II (Cat: R6934-01, Omega, Norcross, GA) according to the manufacturer’s instructions. Fourteen notable DEGs (Table 1) from the RNA-seq analysis were selected for quantitative real time PCR (qRT-PCR) verification in RPE-choroid-sclera of normal and fibrosis eyes collected 20 days after the second laser (*n* = 8). The expression levels of myofibroblast signature genes (*col1a1, FN1, Acta2*) and macrophage activation related genes (Table 1) in BMDMs were also evaluated by qRT-PCR. 1ug of total RNA was used to synthesize cDNA using the PrimeScript RT Reagent Kit (Cat: 6110A, Vazyme Biotech, Nanjing, China). Quantitative real-time PCR was performed in a total of 10ul mixture solution using the LightCycler 96 (Roche, Basel, Switzerland). Each 10 µl reaction mixture contains 5ul SYBR GREEN PCR MasterMix (Cat: Q711-02, Vazyme Biotech), 1uM primers and diluted cDNA. GAPDH was used as a housing-keeping gene. The primers referenced in this study were designed using the NCBI Primer BLAST system and purchased from TSINGKE (Changsha, Hunan, China). The primer sequences are detailed in Table 1.

**Table 1:** Primer sequences of mouse genes for quantitative RT-PCR

Gene	Forward	Reverse
*Mmp12*	CCTGCTTACCCCAAGCTGAT	ATGTTTTGGTGACACGACGG
*Aplnr*	CTGTGCTGGATGCCTTACCA	GCCCGGAAGAATAACTGGCT
*Chil1*	GTCCTGATGCTGCTCCAGA	GGTTGGATGGCGTCTGGTAA
*Jund*	TCTACGCCAACCTGAGCAGT	CGTTCTTGCGTGTCCATGTC
*Smarcd3*	TGACAGATGTGGCAGGGAAC	GCGCTGCTGGATCTTACAGT
*Ctss*	CCACGCTGCCATCAGAAGAT	CCAGATGAGACGCCGTACTT
*Lyz2*	GGAATGGCTGGCTACTATGGA	ACCCATGCTCGAATGCCTTG
*Clec7a*	CTCCATCTTCACCTTGGAGGC	TTGTGTCGCCAAAATGCTAGG
*Itgb2*	CCAGGAATGCACCAAGTACAAAGT	AGTGAAGTTCAGCTTCTGGCAC
*Timp2*	GGATGAGTGCCTCTGGATGG	ACGGGTCCTCGATGTCAAGA
*Nr4a1*	GCACAGCTTGGGTGTTGATG	AGCCATGTGCTCCTTCAGAC
*C1qb*	GCTGGAGACCTTGGTGAGTT	TCGAAGCGAATGACCTGGTT
*CD68*	TCTGATCTTGCTAGGACCGC	TCATCGTGAAGGATGGCAGG
*C3ar1*	TACACTGAACGCTGACGCTT	TGGTTATTGCCATCAGCGGT
*Col1a1*	CTGGCGGTTCAGGTCCAAT	TTCCAGGCAATCCACGAGC
*Fn1*	GCCGTTAGATGTGCAAGCTG	TGCTGAAGCTGAGAACTAGGC
*Acta2*	GGACGTACAACTGGTATTGTGC	TCGGCAGTAGTCACGAAGGA
*iNOS*	GGCAAACCCAAGGTCTACGTT	TCGCTCAAGTTCAGCTTGGT
*Arg-1*	TTATCGGAGCGCCTTTCTCAA	TGGTCTCTCACGTCATACTCTGT
*Emr1(F4/80)*	TTCCTCGCCTGCTTCTTCTG	TAGCCAAAGGCACAGAGGTG
*Smad-1*	CAGGCAGTTGCTTACGAGGA	TCCGGTTAACGTTGGAGAGC
*Smad-2*	GTATGGACACAGGCTCTCCG	ACCAGAATGCAGGTTCCGAG
*Smad-3*	GCGGTCAAGAGCTTGGTGAA	ACCTGGGGATGGTAATGCAC
*GAPDH*	CTCAGGAGAGTGTTTCCTCGTC	ATGGGCTTCCCGTTGATGAC

### In vivo treatment

To investigate the role of MMP12 in subretinal fibrosis, an MMP12 specific inhibitor MMP408 [20] was used in the two-stage laser-induced mouse model of subretinal fibrosis. Three days after the second laser, mice were randomized into 3 groups, MMP408 treatment group, vehicle (1% dimethyl sulfoxide (DMSO) and 2% Tween 80) treatment group, and control non-treatment group. 5 mice were used in each group. MMP408 (Cat: 444291, Sigma Aldrich) was administered via gavage feeding at 5 mg/kg (150 µl/mouse) twice daily for five consecutive days. Mice in the vehicle group received the same amount of 1% DMSO and 2% Tween 80 for five days. Mice were sacrificed 10 days after the second laser and eyes were collected and processed for immunohistochemistry investigations.

### Isolation and culture of bone marrow cells

Bone marrow cells from normal and subretinal fibrosis mice (20 days after the second laser) were isolated using the protocol previously described [21]. Briefly, the bone marrow cells were flushed from the femur and tibia, red blood cells were removed by Lysis Buffer (Cat: 00-4300-54, eBioscience, San Diego, CA). The cells were cultured in DMEM (Cat: 8121516, Gibco, Grand Island, NY) supplemented with 15% fetal bovine serum (FBS, Cat: 10099141C, Gibco), 20% L929 supernatant and 100 mg/ml primocin (Cat: PML-41-06, Invivogen, San Diego, CA) in 6-well plates at a density of 1.5 × 10^6^ cells/well at 37 °C in 5% CO_2_ incubator. The phenotype of BMDMs was confirmed 4 ~ 5 days after culture using flow cytometry and > 96% of cells were CD11b^+^F4/80^+^. The cells were used in the below MMT study. In addition, the expression of MMP12 in BMDMs was examined by immunocytochemistry and qRT-PCR, and supernatants were used for Luminex multiplex cytokine array assay.

### Induction of macrophage-to-myofibroblast transition and MMP408 treatment

The BMDMs from normal non-fibrosis mice or mice with subretinal fibrosis (20 days after 2nd laser) were treated with 10 ng/ml TGF-β1 (Cat: 7666-MB-005, R&D Systems, Minneapolis, MN) with or without MMP408 (2 nM/ml, 20 nM/ml and 80 nM/ml) for 96 h. 0.08% DMSO was used as vehicle control. The cells were collected for (a) qRT-PCR analysis of myofibroblast (*col1a1, FN1, Acta2*) and macrophage polarization (*iNOS, Arg-1*) genes, Smad genes (*Smad-1, Smad-2, Smad-3)* and macrophage marker F4/80 gene (*Emr1*); and (b) immunocytochemistry of collagen-1 and α-SMA.

### Luminex multiplex cytokine assay

The production of 15 cytokines (CCL2, VEGF, CXCL10, Osteopontin, Angiopoietin-2, uPAR, PDGFα, PDGFβ, IL-1α, IL-1β, IL-10, CD105, EGF, GM-CSF, G-CSF) in BMDM supernatants from normal and subretinal fibrosis mice were measured using the Luminex bead-based assay (Cat: LXSAMSM-15, R&D Systems) according to the manufacturer’s instructions. The total protein concentration of the supernatants was determined by BCA assay (Cat: PC0020, Solarbio, Beijing, China). 50ul of the twofold diluted sample was added to a mixture of colour-coded beads pre-coated with 15 capture antibodies and incubated at room temperature for 2 h. 50ul of biotinylated detection antibodies specific to the analytes of interest were loaded and incubated at room temperature for 1 h. Phycoerythrin (PE)-conjugated streptavidin was added and incubated at room temperature for 30 min. Beads were read using the Luminex 200™ analyzer (Luminex, Austin, TX). The levels of cytokines/chemokines in each sample were normalized by the total protein level of the supernatants.

### Human samples

Human eye samples were obtained from San Diego Eye Bank under an Material Transfer Agreement. The eyes were fixed in 10% formalin and shipped to the Queen’s University Belfast. The study complies with the Declaration of Helsinki and the protocol was approved by the Ethical Review Board of Queen’s University Belfast. The samples were then wax embedded and processed for immunohistochemistry detailed previously by us [22].

### Immunohistochemistry

Human eye sections were de-paraffinized, rehydrated. Antigen retrieval was performed by boiling slides in citrate buffer (0.05% citraconic acid, pH 7.4, Sigma-Aldrich) for 30 min. The samples were then blocked with 5% BSA (Cat No: A380310G, Sigma-Aldrich) and processed for primary (rabbit anti-MMP12, 1:25, Cat No: PA5-13181, ThermoFisher Scientific; goat anti-Iba-1, 1:100, Cat No: ab5076, Abcam) and secondary (Alexa Fluor 594 AffiniPure Donkey anti-rabbit IgG(H + L), Alexa Fluor 488 AffiniPure Donkey anti-goat IgG (H + L), all in 1:200, Jackson ImmunoResearch Europe Ltd, Ely, UK) antibodies incubation using the protocol detailed below.

Mouse eyes enucleated at 5, 10 and 20 days post the second laser were fixed in 2% paraformaldehyde (PFA) for 4 h at room temperature and processed for immunohistochemistry of cryosections or RPE/choroid flatmounts. For cryosection staining, the eyes were embedded in an optimum cutting temperature compound (OCT, Sakura, CA) and cryosectioned with 10 µm thickness. The sections were blocked with 10% goat serum, permeabilized with 0.1% Triton X-100 for 1 h, followed by primary antibody incubation at 4 °C overnight. The antibodies include (a) rabbit anti-MMP12 (1:200, Cat: 22989-1-AP, Proteintech, Wuhan, China); (b) rabbit anti-collagen-1 (1:200, Cat: ab34710, Abcam, Cambridge, UK); and (c) rat anti-F4/80 (1:200, Cat: ab6640, Abcam). After thorough washes, samples were incubated with Alexa Fluor 594 goat anti-rabbit IgG (1:200, Cat. No: A11012, Invitrogen) and Alexa Fluor 488 donkey anti-rat IgG (1:200, Cat. No: A48269, Invitrogen) for 2 h at room temperature.

The RPE/choroid flatmounts were blocked with 10% goat serum, and permeabilized with 1% triton X-100 for 2 h at room temperature. The samples were processed for primary (rabbit anti-collagen-1 and rat anti-F4/80) and secondary (Alexa Fluor 594 goat anti-rabbit IgG and Alexa Fluor 488 donkey anti-Rat IgG) antibodies incubation as the protocol described above.

The sections/flatmounts were counter-stained with 4′,6-diamidino-2-phenylindole (DAPI, Cat: D8200, Solarbio) to illustrate cell nuclei. All samples were imaged using the Zeiss LSM 880 Confocal Microscope (Zeiss, Braunschweig, Germany).

### Immunocytochemistry

BMDMs with/without TGFβ and MMP408 treatment were fixed and permeabilized with pre-cold methanol on ice for 15 min. The samples were then incubated with rabbit anti-MMP12 (1:400, Proteintech) and rat anti- F4/80 (1:400, Abcam), or rabbit anti-collagen-1 (1:200, Abcam) and mouse anti-αSMA (1:400, Cat: ab7818, Abcam) at 4 °C overnight, followed by secondary antibodies Alexa Fluor 594 goat anti-rabbit IgG (1:500, Invitrogen) and Alexa Fluor 488 donkey anti-Rat IgG (1:500, Invitrogen) and DAPI (Cat: D8200, Solarbio) for 1 h at room temperature. All samples were imaged using the Zeiss LSM 880 Confocal Microscope (Zeiss).

### Image analysis

The fluorescence intensity of MMP12 was measured using the ZEN Lite software (Zeiss). The fluorescence intensity of the outer nuclear layer was used as a background reference. The levels of MMP12 expression in the choroid and lesion site of subretinal fibrosis were calculated by subtracting the fluorescence intensity of background fluorescence from the fluorescence intensity of choroid or fibrotic lesion. Four to five images (20 × objective lens) were taken for each section and 3 to 5 sections (at least 100 µm apart) were used in each eye. The collagen-1^+^ lesion area and the total number of F4/80^+^ or F4/80^+^collagen-1^+^ cells in RPE-choroid flatmount were investigated in each group. Multiple images were taken from each flatmount to include all F4/80^+^ macrophages (which were often inside and around the fibrotic lesions). The FIJI ImageJ software was used to identify F4/80^+^DAPI^+^ or F4/80^+^collagen-1^+^DAPI^+^ cells. The number of cell nuclei (DAPI^+^) in F4/80^+^ or F4/80^+^collagen-1^+^ area was used to quantify F4/80^+^ or F4/80^+^collagen-1^+^ cells. The analysis was conducted by two independent researchers.

For evaluation of αSMA^+^collagen-1^+^cells in BMDM cultures, three images (10 × objective lens) were taken randomly from each well in 48-well plates. The percentage of αSMA^+^collagen-1^+^cells to the total number of BMDMs was calculated.

### Human RPE/choroid transcriptome datasets analysis

We used the human AMD RPE/choroid transcriptome datasets (ID: GSE135092) from the NCBI GEO database to analyze MMP12 mRNA expression in AMD eyes. The authors conducted bulk RNA sequencing in the RPE-choroid of human AMD eyes [23]. The gene expression in the datasets was quantified by HTSeqGenie as reads FPKM and normalized by DESeq2 [23]. We selected samples in which the information on the location of the tissue (macula vs non-macula) and age of donor was available, including 13 macular RPE/choroid samples and 10 non-macular RPE/choroid samples from AMD donors, and 33 macular RPE/choroid samples and 36 non-macular RPE/choroid samples from age-matched non-AMD donors (age ranges from 70 to 93 years). We analyzed the FPKM expression of MMP12 (ENSG00000262406) in the four groups.

### Statistical analysis

GraphPad Prism (Version 8, GraphPad Software, San Diego, CA) was used for statistical analysis. The difference between two groups was conducted using the Student *t* test or Mann–Whitney test (when sample sizes were less than 10). One-way ANOVA was used when comparing multiple groups followed by Tukey’s multiple comparisons for post hoc test. Kruskal–Wallis multiple comparisons test was used when sample sizes were less than 10. Statistical significance was defined as *p* < 0.05.

## Results

### RNA-seq transcriptome of RPE-choroid from mice with subretinal fibrosis

**Fig. 1:**
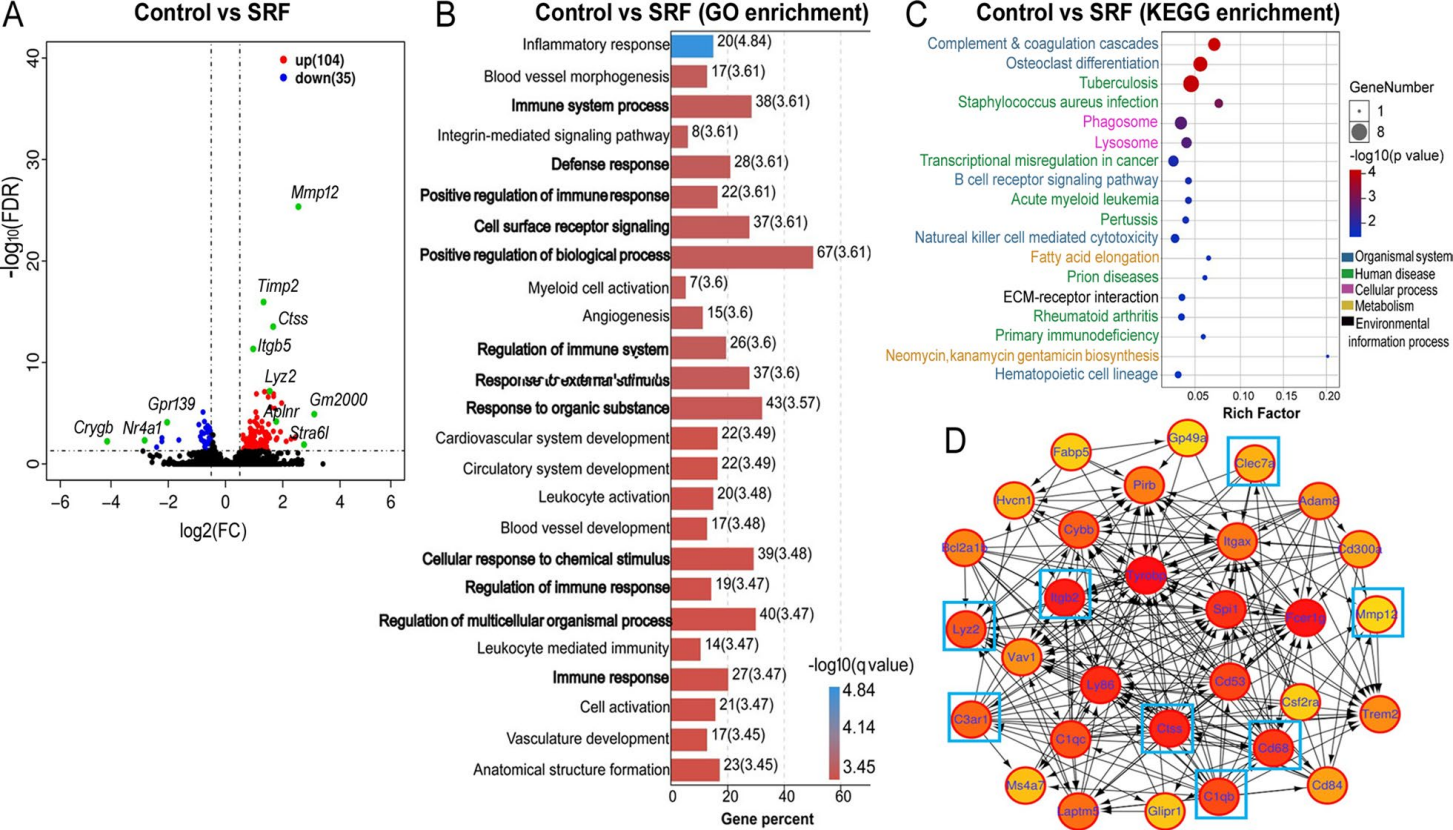
RNA-seq transcriptome of RPE/choroid from control and subretinal fibrosis mice. RNA-seq was conducted in RPE/choroidal tissue from control and subretinal fibrosis mice. The differentially expressed genes (DEGs) were identified as FDR < 0.05, log2(fc) ≥ 0.5, and count > 50 in at least 2 samples. **A** Volcano plot showing the comparison of DEGs from control and subretinal fibrosis mice. The green dots represent the most significantly regulated genes. *n* = 3 mice in each group. **B** The top 25 enriched functions from GO analysis of DEGs. Bold font indicating the significantly enriched GO terms that *Mmp12* is involved in. Numbers beside the bar indicate the number of DEG and q value (in bracket). **C** DEG enriched pathways from KEGG analysis. **D** The network image shows the top 30 highest degree genes calculated by the Cytohubba plugin of Cytoscape. The eight genes (blue boxes) were selected for further qRT-PCR verification

In our RNA-seq study, the clean data of all samples accounted for more than 98.5% of the raw data, and > 91% of raw data had sequenced bases with quality values of Q30. In total, we obtained 15,663 gene features from our sequencing study. There were 139 differentially expressed genes (DEGs) in RPE-choroid from subretinal fibrosis eyes compared to that from the control eyes (fold change log2(fc) ≥ 0.5, FDR < 0.05). Of the 139 DEGs, 104 were up-regulated and 35 were down-regulated (Fig. 1A, Additional file 1: Table S1). The 20 most significantly up-regulated genes are related to cell membrane-extracellular matrix interaction and angiogenesis (*Mmp12, Madcaml, GM49339, Aplnr, Smarcd3, Bcan*) and immune response (*Mmp12, Il7r, Rfxap, Atp6v02d, Chil1, Jund, Dexi, Ctss, Tlr13*) (Table 2, Fig. 1A). Whereas the top 20 down-regulated DEGs were related to the regulation of cell growth (*Nr4a1, Egr3, Spry2, Sik1, Map6d1*), neuronal development (*Crygb, Gpr139, Arc, Rrh, Rd3l, Htr3a*) and immune response (*Gzmm, Ermap*) (Table 2, Fig. 1A).

**Table 2:** Top 20 up- and down-regulated DEGs in subretinal fibrosis mice

Symbol	log2(fc)	FDR	Description of function
Up-regulated DEGs			
*Gm2000*	3.098	0.00001	Translation
*Stra6l*	2.741	0.01272	Retinol transport and vitamin A import
*Mmp12*	2.547	0.00000	Tissue remodelling
*Aard*	2.428	0.00277	Development
*Madcam1*	2.289	0.00350	Cell–matrix adhesion
*Gm49339*	2.119	0.00595	Neutrophil homeostasis
*Atp6v0d2*	1.959	0.00000	Ion transport
*Il7r*	1.918	0.00069	Cytokine receptor activity
*Gm13889*	1.798	0.00279	Data not shown (predicted gene)
*Aplnr*	1.771	0.00007	Angiogenesis
*Rfxap*	1.768	0.00399	Positive regulation of transcription
*Chil1*	1.715	0.00000	Chitin binding activity
*Jund*	1.678	0.00000	Cells protection from senescence and apoptosis
*Smarcd3*	1.676	0.00000	Heart morphogenesis, neural retina development
*Dexi*	1.669	0.00062	Protein binding
*Ctss*	1.663	0.00000	Collagen binding
*Mpnd*	1.579	0.00000	Chromatin remodeling
*Lyz2*	1.543	0.00000	Lysozyme activity
*Bcan*	1.539	0.00277	Calcium ion binding
*Tlr13*	1.501	0.00134	Protein binding activity and rRNA binding activity
Down-regulated DEGs			
*Crygb*	− 4.135	0.00595	Eye development
*Nr4a1*	− 2.825	0.00481	DNA-binding transcription activator activity
*Egr3*	− 2.405	0.02232	DNA-binding transcription activator activity
*Gm20075*	− 2.225	0.00614	Data not shown(predicted gene)
*Arc*	− 2.222	0.00273	mRNA binding activity
*Gpr139*	− 2.039	0.00008	Protein binding activity, neuropeptide receptor activity
*Sik1*	− 1.630	0.00435	ATP binding activity,enzyme binding activity
*Gzmm*	− 0.930	0.00013	T cell mediated cytotoxicity
*Map6d1*	− 0.878	0.00205	Calmodulin binding activity,microtubule binding activity
*Krt23*	− 0.841	0.00426	Structural molecule activity
*Spry2*	− 0.833	0.00076	Negative regulation of cell differentiation
*Adora2b*	− 0.785	0.00001	G protein-coupled receptor activity
*Fam13a*	− 0.764	0.00587	GTPase activator activity
*Ermap*	− 0.759	0.00098	Signaling receptor binding activity
*Rrh*	− 0.736	0.00007	Protein-coupled photoreceptor activity, Phototransduction
*Rd3l*	− 0.733	0.00824	Visual perception
*Htr3a*	− 0.729	0.02029	Protein binding activity
*Dusp6*	− 0.714	0.00669	Negative regulation of protein phosphorylation
*Upk3b*	− 0.701	0.01424	Negative regulation of glucose import
*Mogat1*	− 0.698	0.04907	Lipid metabolic process

To understand the biological functions affected by the 139 DEGs, we conducted Gene Ontology (GO) enrichment analysis. In total, 268 GO terms were significantly enriched (q value < 0.05, Additional file 1: Table S2), including 250 in biological processes, 15 in cellular components, and 3 in molecular functions. The top 25 enrichment GO terms belonged to biological processes (Fig. 1B), and the majority of them were related to inflammation, blood vessels/cardiovascular development and angiogenesis (Fig. 1B). Ninety of the 139 DEGs contributed to the top 25 enriched terms, indicating a high degree of crosstalk between these terms. Of interesting to note, one of the most significantly upregulated genes, *Mmp12*, contributed to 12 of the top 25 GO terms (Fig. 1B, bold font).

To gain insights into the molecular pathways affected by the DEGs, we further conducted KEGG enrichment analysis and identified 18 significantly enriched pathways (Fig. 1C). Twelve of them related to immune responses including the complement and coagulation cascades (Fig. 1C). Other enriched pathways include fatty acid elongation, neomycin/kanamycin biosynthesis (D-glucose metabolism), and ECM-receptor interaction (Fig. 1C).

To further understand the genes associated with retinal fibrosis, we constructed a PPI network using the STRING database with the139 DEGs, and obtained 136 nodes and 312 edges with a confidence score > 0.4. Figure 1D showed the 30 highest degree genes calculated by the Cytohubba plugin of Cytoscape. The darker the colour, the higher degree of connectivity of the nodes. The eight genes in blue boxes (*Mmp12, CD68, Itgb2, Ctss, C1qb, C3ar1, Clec7a, Lyz2*) were selected for further qRT-PCR verification (Fig. 1D).

**Fig. 2:**
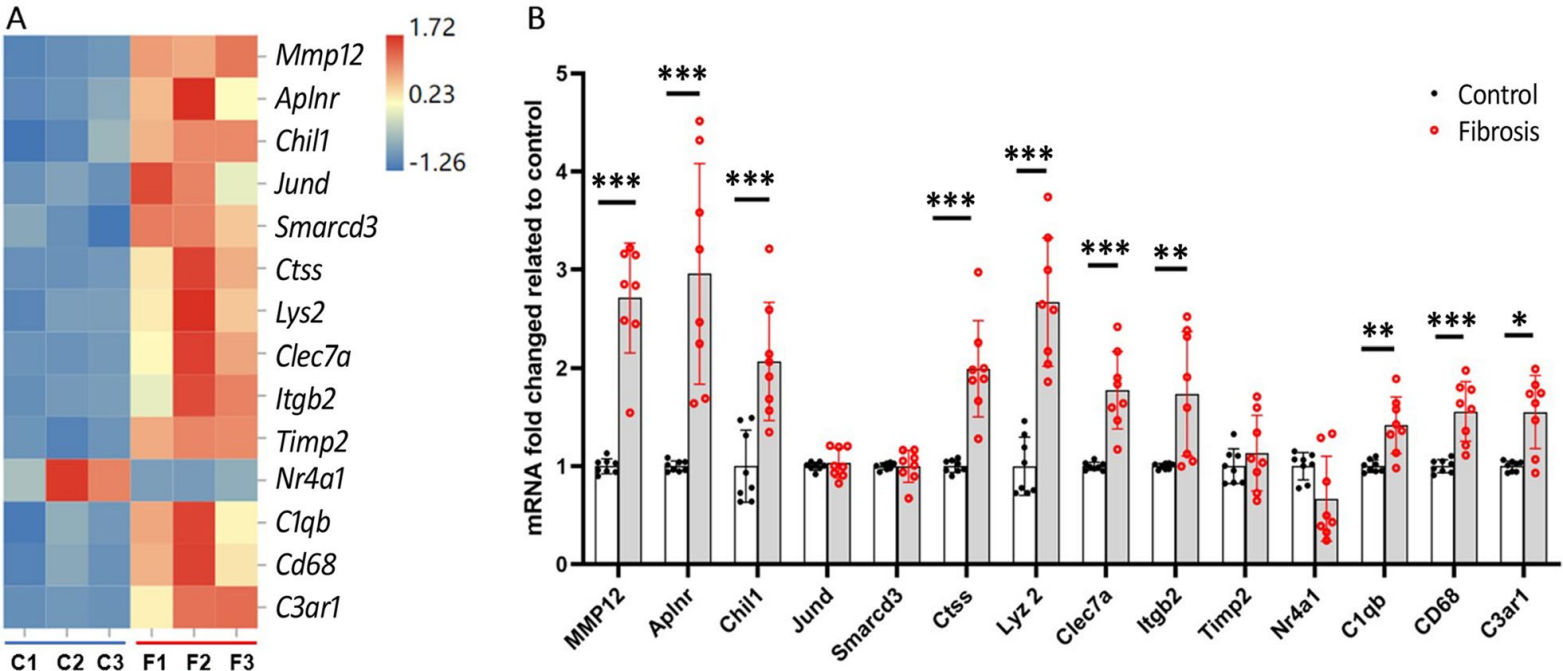
Verification of selected DEGs in RPE-choroid from control and subretinal fibrosis mice via qRT-PCR. **A** Heat map showing the expression levels of selected DEGs in RPE-choroid from control and subretinal fibrosis eyes in RNA-seq analysis. C = control, F = fibrosis. Data showing as FPKM, *n* = 3 mice. **B** qRT-PCR analysis of selected 14 notable DEGs expressions in RPE-choroid from control and subretinal fibrosis eyes. Mean ± SD, *n* = 8 mice, *p < 0.05, **p < 0.01, ***p < 0.001. Mann–Whitney test

The expression levels of 14 DEGs (including the 8 DEGs shown in blue boxes in Fig. 1D) in control and fibrotic tissues were further analyzed by qRT-PCR (Fig. 2A). Ten were confirmed (*Mmp12, Aplnr, Chil1, Ctss, Lyz2, CD68, Itgb2, C1qb, C3ar1, Clec7a,*) and 3 were not (*Jund, Smarcd3, Timp2*) (Fig. 2B). The remaining one, *Nr4a1* was downregulated in the RNA-seq analysis (Fig. 2A) and qRT-PCR showed a trend, but statistically insignificant reduction (*p* = 0.058, Fig. 2B).

### MMP12 expression in subretinal fibrotic lesion

**Fig. 3:**
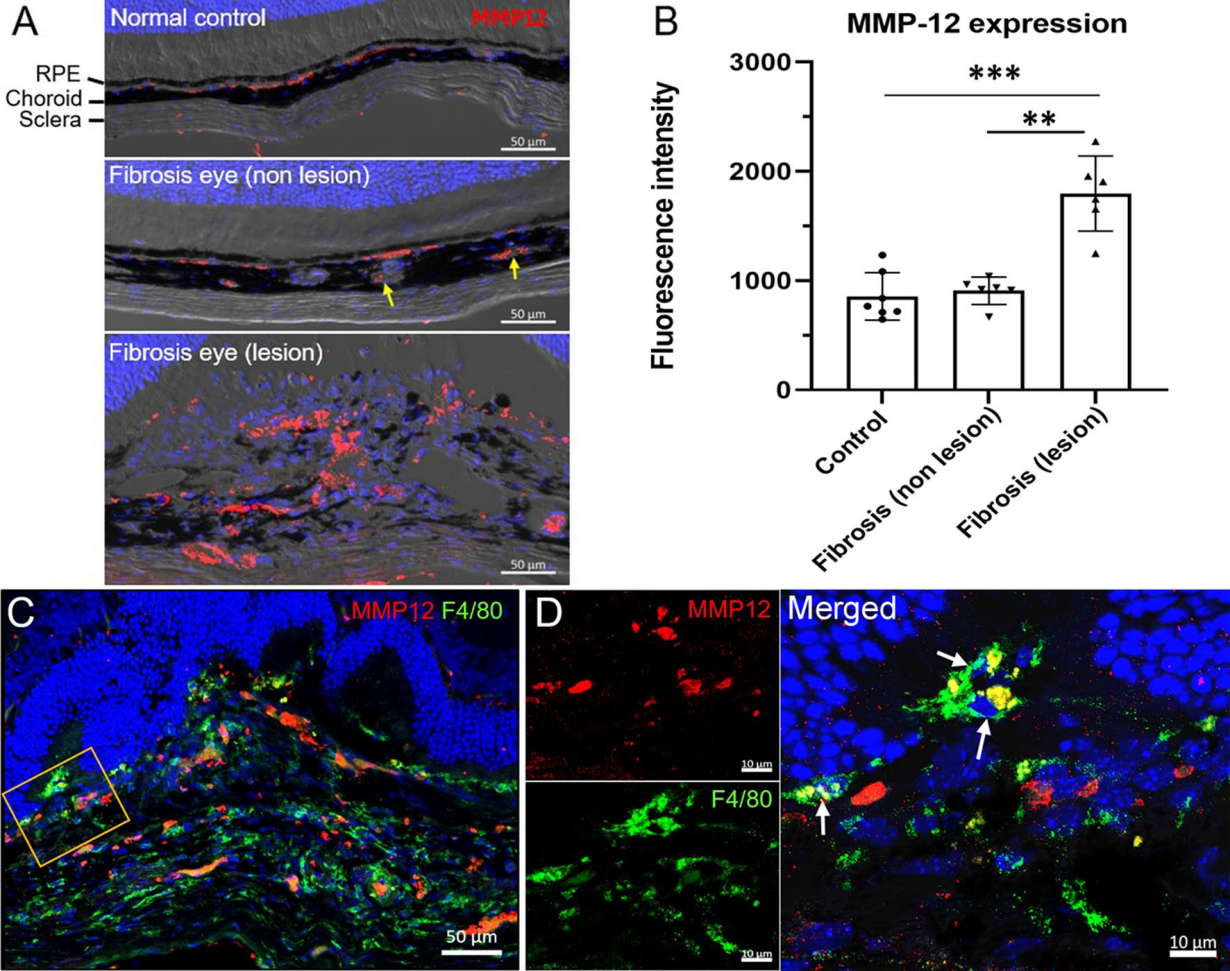
MMP12 expression in mouse eyes with/without subretinal fibrosis. **A** Representative confocal images showing MMP12 (red) expression in the choroid/sclera of eyes from normal and subretinal fibrosis (5 days after the second laser) mice. Yellow arrows indicate MMP12^+^ cells in choroidal blood vessels. **B** Dot/bar figure showing fluorescence intensity of MMP12 in the choroid of normal eyes, non-lesion site and fibrotic lesions of fibrosis eyes. Mean ± SD, *n* = 6–7 eyes. **p < 0.01, ***p < 0.001, Kruskal–Wallis with Dunn’s multiple comparisons test. **C** Confocal image from a subretinal fibrosis eye showing MMP12 (red) and F4/80 (green). A high magnification view of the yellow rectangle area, including each channel, is shown in (**D**). Arrows indicate F4/80^+^MMP12^+^ cells

Among the hub genes identified from the RNA-seq analysis, *Mmp12* caught our attention due to its known roles in inflammation-mediated lung and liver fibrosis [24–26]. Confocal microscopy detected discrete MMP12 expression in the choroid and occasionally the sclera of normal mouse eyes (Fig. 3A) as well as in the non-lesion area of the choroid from fibrosis eyes (Fig. 3A). MMP12^+^ cells were also observed in choroidal blood vessels, presumably circulating immune cells (arrows, Fig. 3A). Extensive MMP12 expression was observed in the subretinal fibrosis lesion area (Fig. 3A, B). The expression levels of MMP12 in the choroid of normal mice and the non-lesion sites of fibrosis eyes were comparable (Fig. 3B). Dual staining of MMP12 and F4/80 showed that 20.2 ± 1.6% (mean ± SD, *n* = 6) of F4/80^+^ macrophages in fibrotic lesion co-expressed MMP12 (Fig. 3C, D), suggesting that macrophages may be a major source of MMP12 in subretinal fibrosis**.**

### MMP12 expression in bone marrow-derived macrophages (BMDMs)

**Fig. 4:**
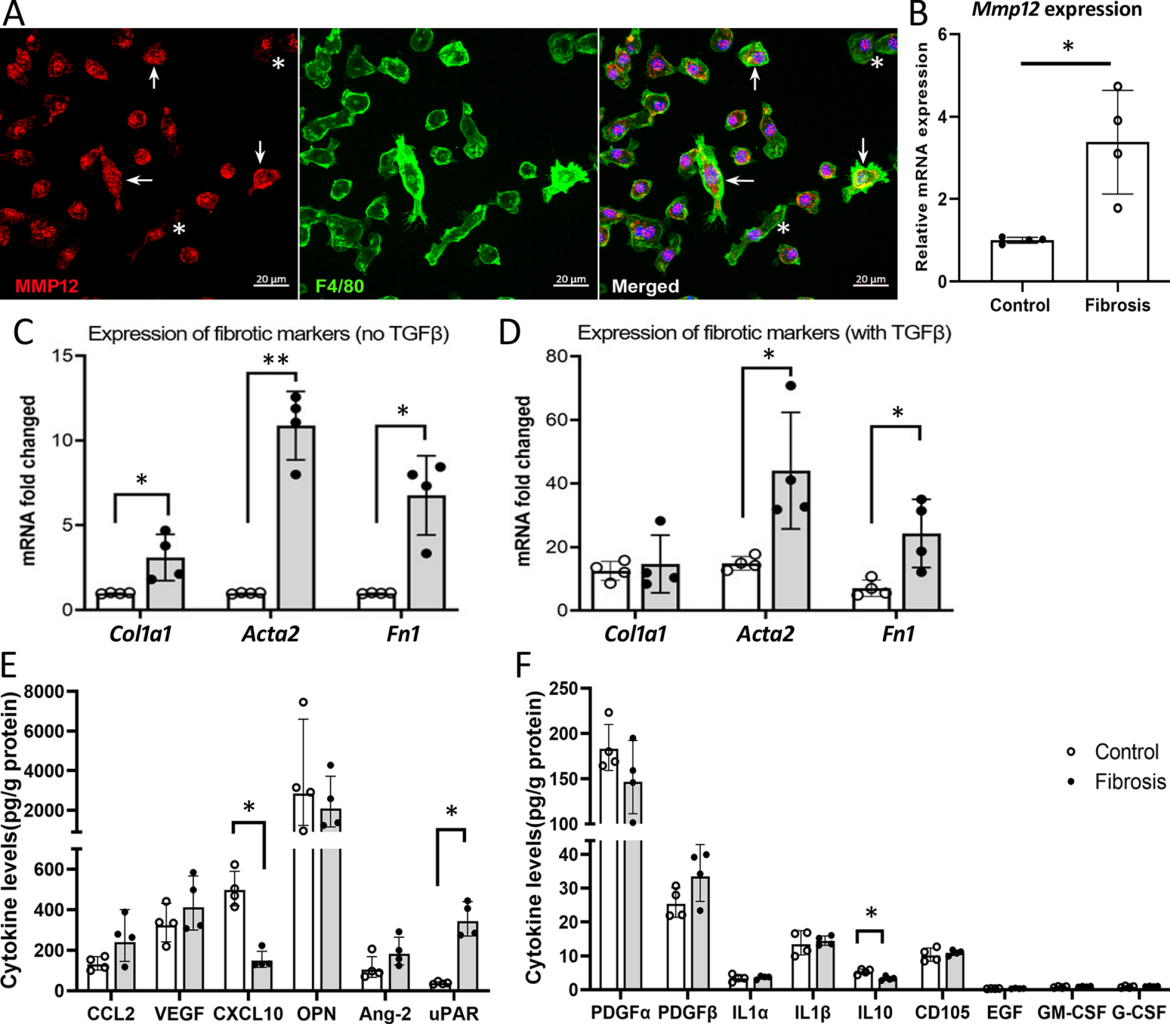
The expression of MMP12 and pro-fibrotic markers in bone marrow-derived macrophages (BMDMs) from control and subretinal fibrosis mice. BMDMs were cultured with/without 10 ng/ml recombinant TGF-β1 for 96 h, the cells were collected for immunocytochemistry or qRT-PCR, and supernatants were used for Luminex multiplex cytokine assay. **A** BMDMs from normal mice were stained for MMP12 (red) and F4/80 (green), imaged by confocal microscopy. Arrows indicating MMP12^high^ in F4/80^+^ cells. Asterisks indicating MMP12^low^ in F4/80^+^ cells. **B** qRT-PCR analysis of *Mmp12* expression in BMDMs from control and subretinal fibrosis mice (20 days after the second laser). **C**, **D** qRT-PCR analysis of fibrotic marker genes (*Col1a1, Acta2, Fn1*) in untreated BMDMs (**C**) and TGFβ1 treated BMDMs (**D**) from control and subretinal fibrosis mice. **E**, **F** Luminex bead-based assay of the production of pro-fibrotic cytokines by BMDMs from normal and subretinal fibrosis mice. Mean ± SD, *n* = 4 mice, *p < 0.05, **p < 0.01, Mann–Whitney test

To understand how macrophage-derived MMP12 may contribute to subretinal fibrosis, we examined the MMP12 expression in BMDMs. Immunocytochemistry detected MMP12 in almost all F4/80^+^ BMDMs although the expression level varies in different cells (MMP12^hi^ in arrows, MMP12^low^ in asterisks, Fig. 4A). Quantitative RT-PCR showed that the expression levels of *Mmp12* in BMDMs from mice with subretinal fibrosis were significantly higher than those from control mice (Fig. 4B). Interestingly, the basal levels of collagen-1 (*Col1a1*), αSMA (*Acta2*) and fibronectin (*Fn1*) in BMDMs from subretinal fibrosis mice were also significantly higher than those from control mice (Fig. 4C). TGFβ1 treatment reduced *iNOS* (*NOS2*), but increased *Arg-1* expression (Additional file 1: Fig. S1A) accompanied by higher levels of Smad1-3 expression (Additional file 1: Fig. S1B) in BMDMs. The treatment did not affect *Emr1* (F4/80) expression (Additional file 1: Fig. S1A). In line with the in vitro TGFβ-treatment study, the expression of *iNOS* (Additional file 1: Fig. S1C) was reduced and the expression of *Arg-1* was increased in the RPE/choroid from subretinal fibrosis eyes (Additional file 1: Fig. S1D). TGFβ treatment also increased the expressions of myofibroblast markers collagen-1 (*Col1a1*), αSMA (*Acta2*) and fibronectin (*Fn1*) in BMDMs (Fig. 4D), and the increment levels of *Acta2* and *Fn1* were significantly higher in cells from subretinal fibrosis mice compared to those from controls (Fig. 4D). Multiplex cytokine array assay showed that BMDMs from subretinal fibrosis mice produced significantly higher levels of uPAR compared to BMDMs from control mice (Fig. 4E), and a trend of increase in CCL2 and PDGFβ (Fig. 4E, F), whereas the production of CXCL10 and IL-10 was significantly lower in BMDMs from subretinal fibrosis mice (Fig. 4E, F). These results suggest that macrophages from subretinal fibrosis mice were pro-angiogenic and pro-fibrotic.

### The effect of MMP12 inhibition on macrophage-to-myofibroblast transition (MMT)

**Fig. 5:**
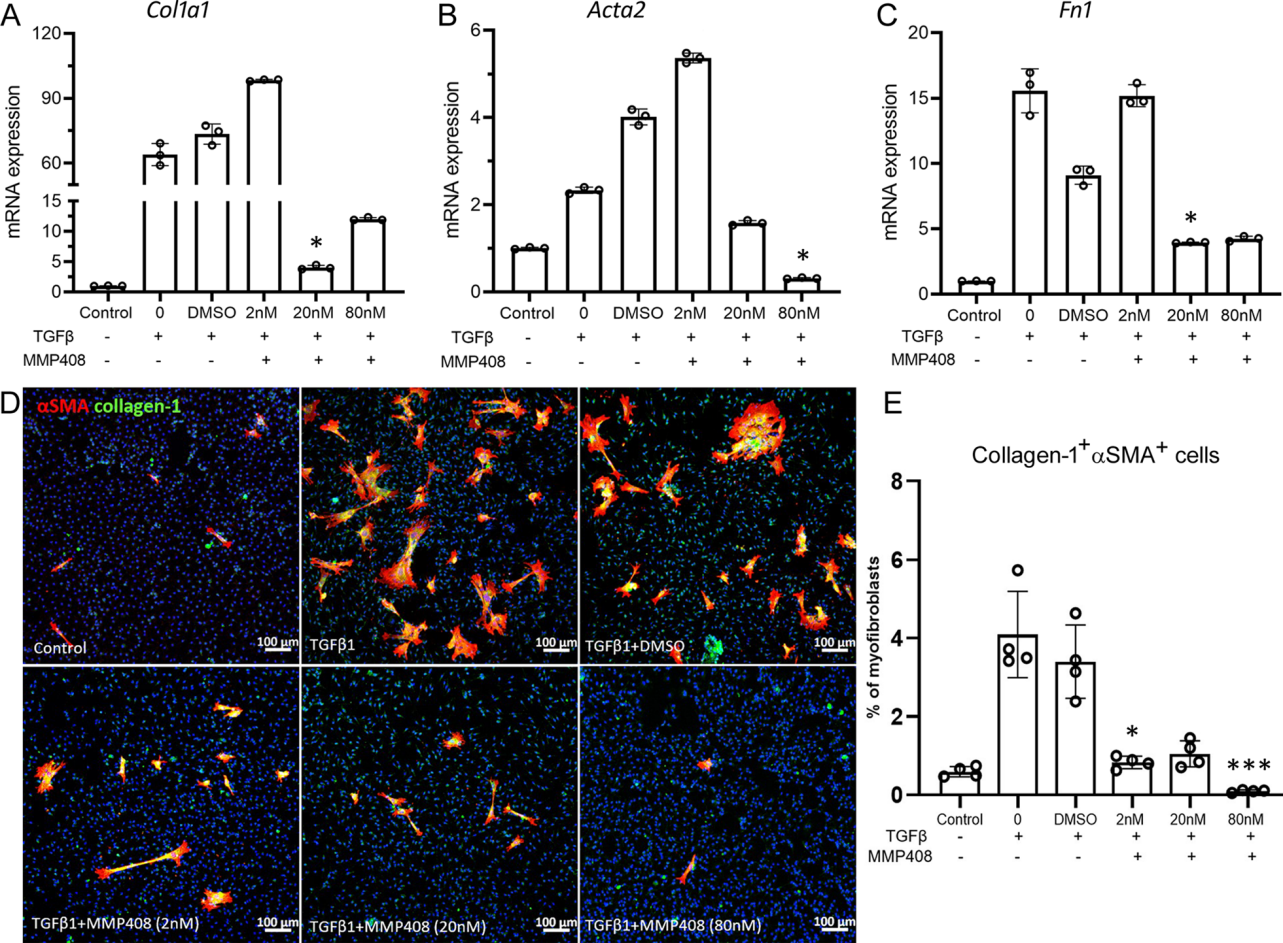
The effect of MMP408 on macrophage-to-myofibroblast transition (MMT). BMDMs from normal mice were treated with TGFβ1 (10 ng/ml) with or without different concentrations of MMP408 (2 nM/ml, 20 nM/ml and 80 nM/ml) or DMSO for 96 h. Cells were collected for qRT-PCR or immunocytochemistry. **A**–**C** The mRNA expression levels of *Col1a1* (**A**), *Acta2* (**B**) and *Fn1* (**C**). Mean ± SD, *n* = 3, *p < 0.05 compared with DMSO group. Kruskal–Wallis with Dunn’s multiple comparisons test. **D** Representative confocal images of BMDMs stained for α-SMA (red) and collagen-1(green) from different treatment groups. Scale bar = 100 µm. **E** Dot/bar figure showing the percentages of collagen-1^+^ α-SMA ^+^ cells in different groups. Mean ± SD, *n* = 4, **p < 0.01, ***p < 0.001. Kruskal–Wallis with Dunn’s multiple comparisons test

Previously, we reported the existence of macrophage-to-myofibroblast transition (MMT) in subretinal fibrosis [22]. To understand if MMP12 is involved in MMT, we used an MMP12 selective inhibitor MMP408 in the MMT study. MMP408 at the concentrations of 20 and 80 nM significantly reduced TGFβ1-induced upregulation of *Col1a1*, *Acta2* and *Fn1* mRNA in BMDMs (Fig. 5A–C). MMP12 at a low concentration (2 nM) appeared to increase the mRNA expression of *Col1a1*, *Acta2* and *Fn1* compared to DMSO treated group (Fig. 5A–C). However, immunocytochemistry showed strong reductions in the percentage of αSMA^+^Collagen-1^+^ cells in all groups treated by MMP408, including the 2 nM group (Fig. 5D, E).

### The effect of MMP12 inhibition on subretinal fibrosis

**Fig. 6:**
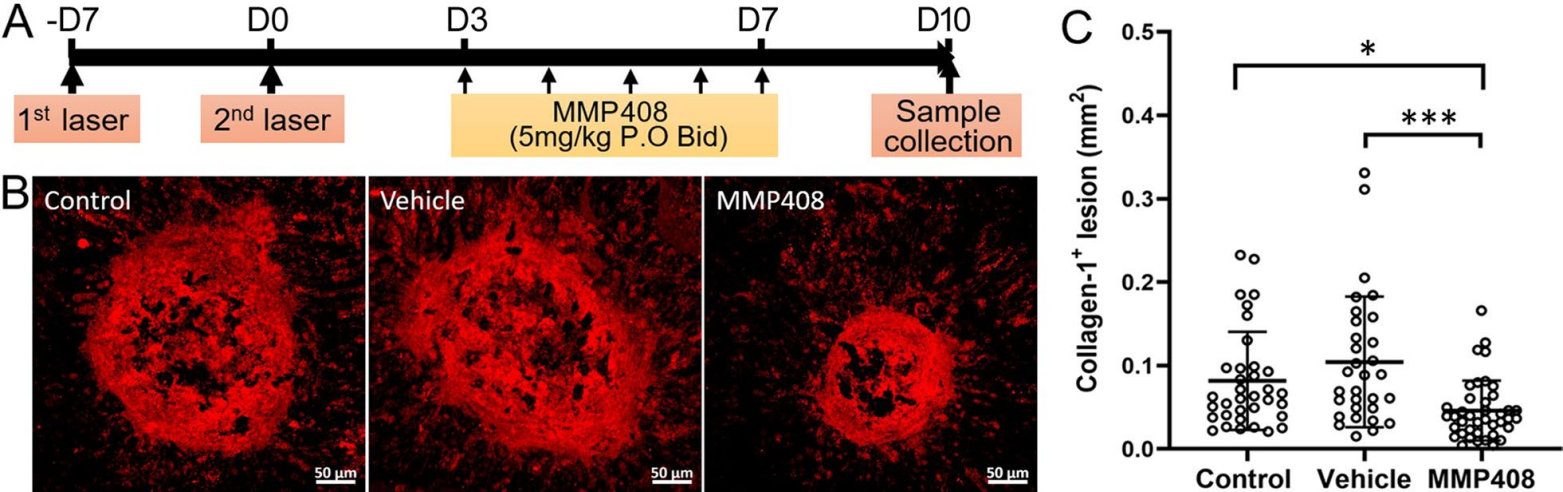
The effect of MMP408 on subretinal fibrosis. **A** Schematic view of the study design. Mice were treated with MMP408 (5 mg/kg twice daily, gavage) or vehicle three days after the second laser for 5 days. Eyes were collected on day 10 for immunohistochemistry. Subretinal fibrosis mice that received no treatment were considered as a treatment-naïve control group. **B** Representative confocal images of RPE/choroid flatmounts stained for collagen-1 from control non-treatment group, vehicle group and MMP408 group. Scale bar = 50 µm. **C** Quantitative analysis of collagen-1^+^ lesion area in different groups. Mean ± SD, *n* = 31–40 lesions per group from 8 to 10 eyes, *p < 0.05, **p < 0.01. One-way ANOVA followed by Tukey’s multiple comparison’s test

Next, we investigated the effect of MMP408 in subretinal fibrosis in our two-stage laser-induced in vivo model. MMP408 was given three days after the second laser daily for 5 days (Fig. 6A). Eyes were collected 10 days after the second laser (Fig. 6A). MMP408 significantly reduced the size of collagen-1^+^ fibrotic lesions compared with no-treatment controls and vehicle (DMSO) treated mice (Figs. 6B, C).

**Fig. 7:**
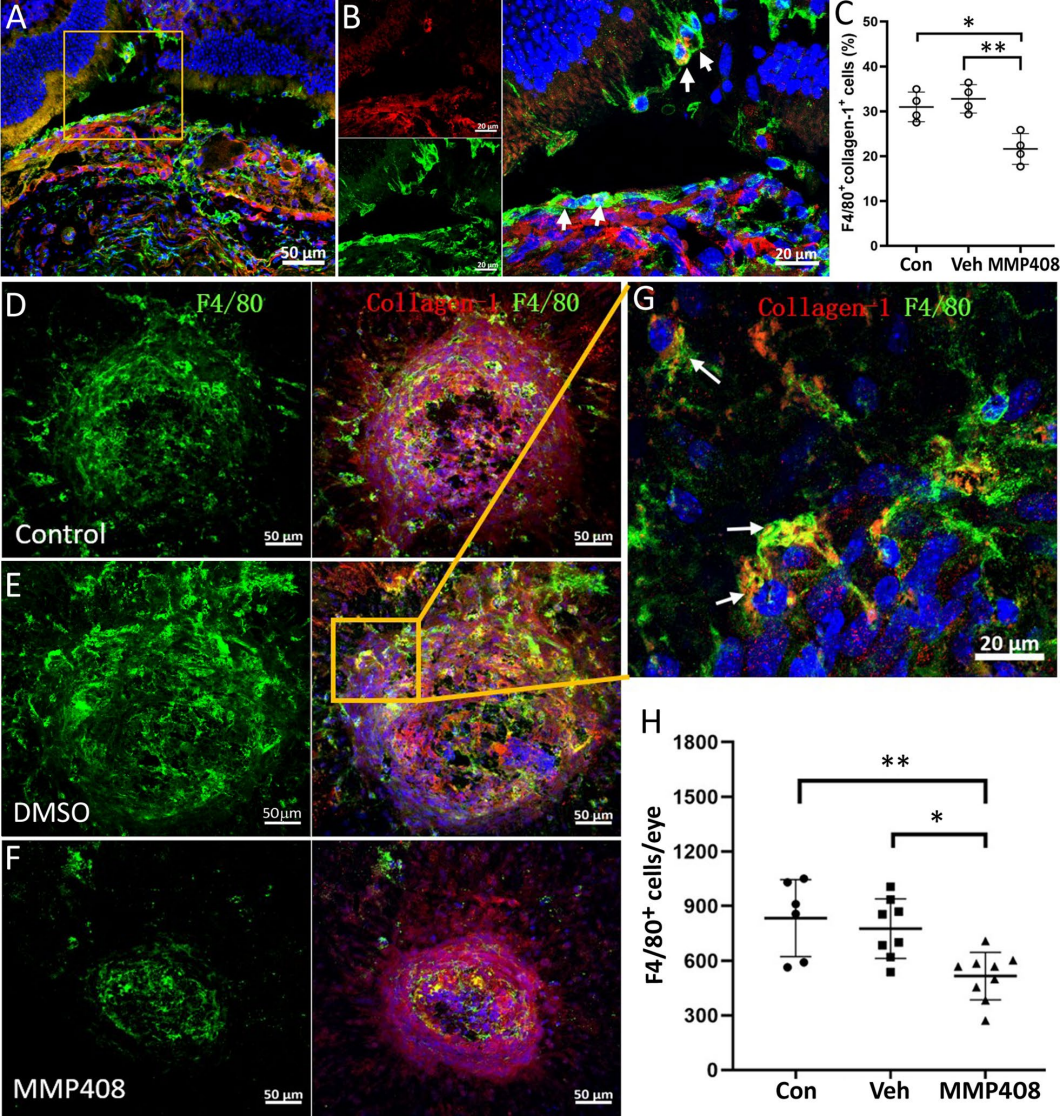
The effect of MMP408 on macrophage accumulation in subretinal fibrosis. **A** Cryosections from subretinal fibrosis eyes were stained for collagen-1 (red) and F4/80 (green) and imaged by confocal microscopy. **B** Magnified area of box in (**A**) showing F4/80^+^MMP12^+^ cells (arrows) inside and around the subretinal fibrotic lesion. **C** The percentage of F4/80^+^Collagen-1^+^ cells among all F4/80^+^ macrophages. **D**–**H** Representative confocal images of RPE/choroid flatmounts stained for collagen-1 (red) and F4/80 (green) from control non-treatment group (**D**), vehicle (DMSO) group (**E**) and MMP408 group (**F**). Scale bar = 50 µm. **G** Magnified area of box in (**E**) showing F4/80^+^collagen-1^+^ cells (arrows) in the fibrotic lesion. **H** Quantitative analysis of the total F4/80^+^ cells in RPE-choroid flatmount in different groups. Mean ± SD, *n* = 6–9 eyes from 4–5 mice, *p < 0.05, **p < 0.01. Kruskal–Wallis with Dunn’s multiple comparisons test

A large number of F4/80^+^ macrophages were detected inside and around the fibrotic lesions in ocular sections (Fig. 7A–C) and RPE-choroid flatmounts (Fig. 7D–G). We found ~ 31% of F4/80^+^ macrophages co-expressed collagen 1 in control subretinal fibrosis mice and this was reduced to 21% following MMP408 treatment (Fig. 7B, C), suggesting that MMP12 inhibition reduced MMT. The total number of F4/80^+^ cells per RPE-choroidal flatmount in MMP408 treated mice was significantly lower than that of non-treatment controls and vehicle-treated mice (Fig.7H).

### MMP12 mRNA expression in human age-related macular degeneration

**Fig. 8:**
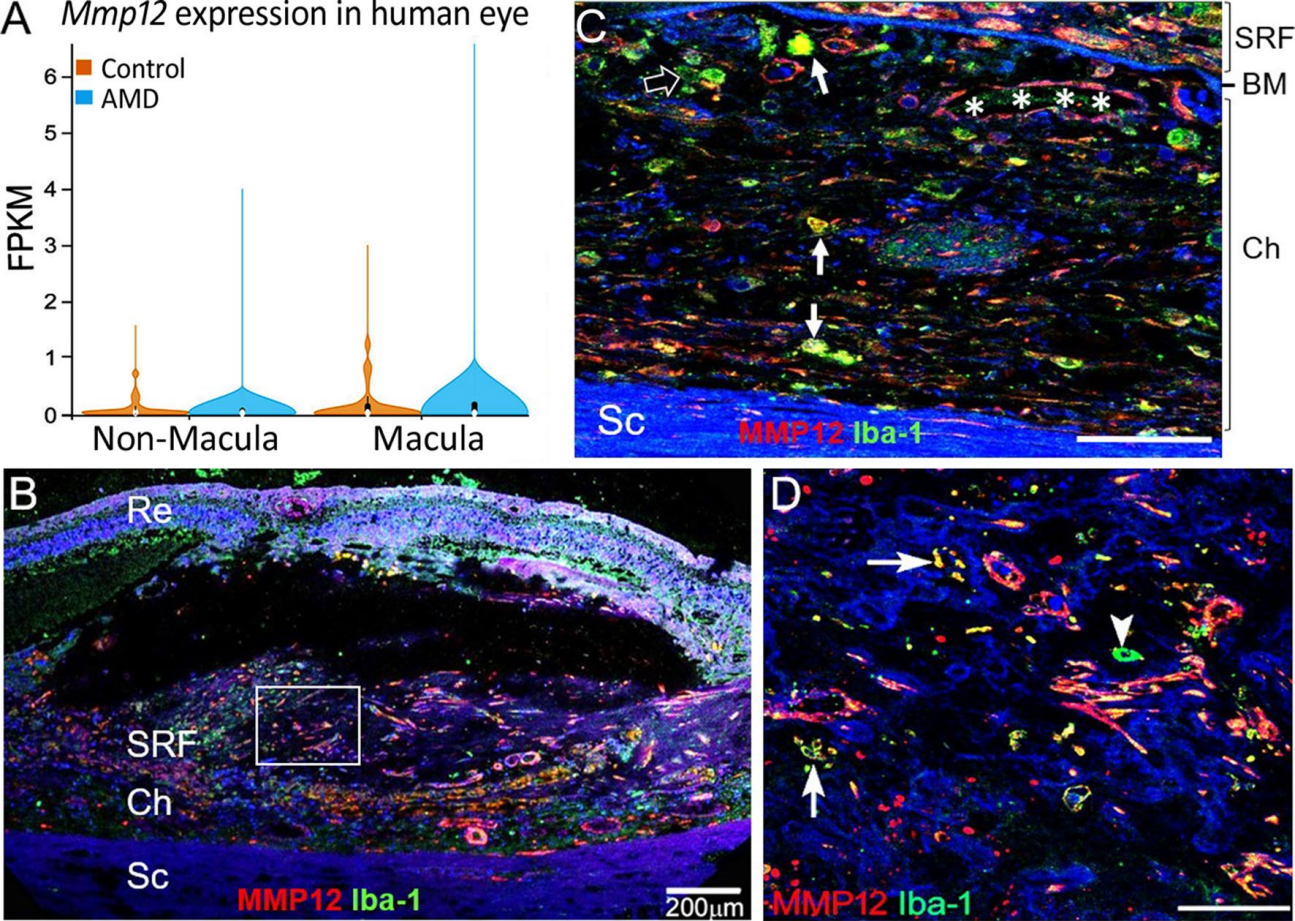
The expression of MMP-12 in human AMD and macular fibrosis. **A** The mRNA expression levels of *Mmp12* in non-macular and macular RPE-choroid from non-AMD controls and AMD (Bulk RNA-Seq data from NCBI GEO database, ID: GSE135092). Mean ± SD, (AMD macula, *n* = 13; AMD non-macula, *n* = 10; non-AMD macula, *n* = 33; non-AMD non-macula, *n* = 36). **B** A confocal image from human nAMD with macular fibrosis showing MMP12 (red) and Iba-1 (green) in the choroid and subretinal lesion. **C** High magnification image showing Iba-1^+^MMP12^+^ cells (arrows), Iba-1^+^MMP12^−^ cells (open arrow) in the choroid and MMP12^+^ cells in choroidal blood vessels (asterisks). **D** Magnified area of box in (**B**) showing Iba-1^+^MMP12^+^cells (arrows), Iba-1^+^MMP12^−^cells (arrowhead) in the macular fibrotic lesion. Re = retina, SRF = subretinal fibrotic lesion, Ch = choroid, BM = Bruch’s membrane. Scale bars in C and D = 50 µm

Upon analyzing the online AMD sample bulk RNA-seq datasets (GSE135092) [23], we found that *Mmp12* mRNA was detected in both macular and non-macular RPE/choroidal tissues. The expression level of *Mmp12* in AMD macular tissues appeared to be higher than that in non-AMD controls but the difference did not reach statistical significance (*p* = 0.194 by Welch’s t test, Fig. 8A). There was no significant difference in the expression levels of MMP12 in non-macular RPE-choroid specimens between AMD and non-AMD controls (Fig. 8A).

Immunofluorescence staining of Iba-1 and MMP12 in human nAMD eyes with macular fibrosis showed that many Iba-1^+^MMP12^+^ cells in the diseased eye (Fig. 8B) including the choroid (Arrows, Fig. 8C) and subretinal fibrotic lesion (Arrows, Fig. 8D). We also detected Iba-1^+^MMP12^−^ cells in the lesion (open arrow in Fig. 8C, arrowhead Fig. 8D).

## Discussion

In this study, using the RNA-seq technique, we found that inflammatory pathways along with fatty acid synthesis and ECM-receptor interaction pathways are highly enriched in subretinal fibrosis. Our results confirmed the role of inflammation in the development of subretinal fibrosis. Myofibroblast infiltration and activation is a key step for the initiation of macular fibrosis [9]. Inflammatory mediators may recruit and activate fibroblasts and fibrocytes from the choroid and blood circulation. They can also induce EMT/EndoMT in RPE and vascular endothelial cells [9, 18, 27]. We recently reported that macrophages are also a source of myofibroblasts in subretinal fibrosis through MMT [22]. Here, we demonstrate that MMP12 is critically involved in the development of subretinal fibrosis. Mechanistically, we found that MMP12 may participate in the regulation of macrophage phenotype by driving M2 differentiation and promoting MMT.

MMP12 is reported to play an important role in organ fibrosis including the lung [24, 25, 28, 29], liver [30] skin and heart [26]. In the retina, MMP12 deficiency reduced retinal inflammation and pathological angiogenesis in oxygen-induced retinopathy [31]. We found that MMP12 expression was significantly increased in subretinal fibrosis and a substantial amount of MMP12 was detected in F4/80^+^ macrophages, a major cellular source of MMP12. We also found that macrophages from subretinal fibrosis mice expressed higher levels of MMP12 and were pro-angiogenic and pro-fibrotic with or without TGFβ stimulation. TGFβ is known to be able to induce MMT [22, 32], which was confirmed in this study. TGFβ treatment also increased MMP12 expression in macrophages accompanied by induction of an M2-like phenotype evidenced by reduced *iNOS* and increased *Arg-1* expression. This is in line with a previous report, in which the authors showed that TGFβ induced M2-like macrophage polarization through SNAIL-mediated suppression of pro-inflammatory phenotype [33]. We found that blocking MMP12 dose-dependently reduced TGFβ-induced MMT in vitro, suggesting that MMP12 may directly participate in the MMT process. Importantly, we showed that MMP12 is present in human ocular tissue at both mRNA and protein levels, and the fibrotic lesions from nAMD eyes contained a large number of MMP12^+^Iba1^+^ cells. Our results suggest that MMP12^+^ macrophages may play a role in macular fibrosis secondary to neovascular AMD.

Exactly how MMP12 contributes to MMT is not known. Apart from the elastase activity, MMP12 is capable of degrading some proinflammatory mediators such as pro-TNFα [34], IFNγ [35] and CXC and CC chemokines [36]. The removal of these proinflammatory cytokines by MMP12 from the microenvironment of macrophages may promote their differentiation towards M2-like wound healing and pro-fibrotic phenotype. However, the fact that inhibition of MMP12 almost completely abrogated TGFβ-induced MMT (Fig. 5D) suggests that MMP12 may be directly involved in TGFβ signalling. It would be interesting to know whether MMP12 is involved in TGFβ-TGFβR1/R2 interaction and the downstream signalling of the TGFβR1/R2-smad pathway.

In addition to its role in MMT, MMP12 may also contribute to retinal fibrosis through other mechanisms, such as promoting macrophage infiltration and regulation of immune responses. The elastase activity of MMP12 can generate various elastin-derived peptides (EDPs). The main repeating sequence of EDPs, Val-Gly-Val-Ala-Pro-Gly (VGVAPG) hexapeptide can interact with cell surface elastin-receptor complex consisting of elastin-binding protein, cathepsin A, and neuraminidase [37]. EDPs can also interact with the scavenger receptor CD36, galectin-3, and integrin αvβ3 and αvβ5 in tissue and immune cells [37, 38]. EDP-receptor interaction is known to be involved in cell migration, proliferation, apoptosis, inflammation and tissue remodelling [38]. In addition, MMP12 can degrade a spectrum of other extracellular matrix proteins, including type IV collagen, fibronectin, laminin, heparin sulphate and chondroitin sulphate [34, 39–41]. A recent study has shown that MMP12 is critically involved in macrophage transmigration across the intestinal epithelial barrier, probably through the degradation of the basement membrane [35]. Elected levels of MMP12 after ischemic stroke was reported to degrade several tight junction proteins including claudin-5, occludin and ZO-1 [42]. MMP12 may damage the blood barrier and promote macrophage infiltration in our model of subretinal fibrosis. Indeed, blocking MMP12 with MMP408 significantly reduced the number of F4/80^+^ macrophages in our study (Fig. 7) and suppressed subretinal fibrosis (Fig. 6).

## Conclusions

In this study, we have provided evidence support for macrophage-derived MMP12 as an important mediator of subretinal fibrosis. Our result has significant implications in the pathogenesis of macular fibrosis in AMD patients as MMP12 is highly expressed in the diseased eyes. Macrophages are one of the major cell types driving organ fibrosis. Understanding the complete arsenal of macrophages in neovascular AMD allows us to identify and design interventions to prevent or treat macular fibrosis.

## Supplementary Information

**Additional file 1: Figure S1.** (A, B) The expression of macrophage phenotype genes and Smads in BMDMs with/without TGFβ1 treatment. BMDMs from naïve mice were treated with/without 10ng/ml of TGF-β1 for 96 h. Cells were collected for qRT-PCR analysis. (A) The relative mRNA expression levels of *iNOS, Arg-1, Emr1 *(F4/80) in control and TGF-β1 treated BMDMs. (B) Relative mRNA expression levels of *Smad-1, Smad-2, *and* Smad-3* in control and TGFβ1-treated BMDMs. Mean ± SD, n = 3. **p<0.01, ***p < 0.001, Mann-Whitney test. (C, D) The expression of iNOS (C) and Arg-1 (D) in RPE/choroidal tissues from normal (Con) and day 5 and day 10 subretinal fibrosis mice. Mean ± SD, n = 6 eyes. **p< 0.01, Kruskal-Wallis with Dunn’s multiple comparisons test. **Table S1.** The list of differentially expressed genes (DEGs) in RPE-choroid from normal and subretinal fibrosis mice. **Table S2. **The list of significantly enriched Gene Ontology (GO) terms.

## Data Availability

The processed bulk RNA-seq data generated during the current study are available in the GEO repository (Access number: GSE189555).

## Abbreviations

αSMA (Acta2): Alpha smooth muscle actin
BMDMs: Bone marrow-derived macrophages
CNV: Choroidal neovascularization
DEGs: Differentially expressed genes
DMSO: Dimethyl sulfoxide
EMT: Epithelial to mesenchymal transition
EndoMT: Endothelial to mesenchymal transition
EDPs: Elastin-derived peptides
FPKM: Fragments per kilobase of transcript per million mapped reads
GO: Gene ontology
KEGG: Kyoto Encyclopedia of Genes and Genomes
MMT: Macrophage-to-myofibroblast transition
MMP12: Matrix metalloproteinase 12
nAMD: Neovascular age-related macular degeneration
PPI: Protein–protein interaction
qRT-PCR: Quantitative real-time polymerase chain reaction
RPE: Retinal pigment epithelial
RNA-seq: Ribonucleic acid sequencing
TGF-β1: Transforming growth factor beta1
Aplnr: Apelin receptor
Chil1: Chitinase-like 1
Ctss: Cathepsin S
Lyz2: Lysozyme 2
CD68: Cluster of differentiation 68
Itgb2: Integrin beta 2
C1qb: Complement component 1, q subcomponent, beta polypeptide
C3ar1: Complement component 3a receptor 1
Clec7a: C-type lectin domain family 7, member a
