# Supplementary material for: Macrophage elastase (MMP12) critically contributes to the development of subretinal fibrosis
Source: J Neuroinflammation. 2022 Apr 5;19:78. doi: 10.1186/s12974-022-02433-x (PMC8985356; doi:10.1186/s12974-022-02433-x)
Supplement: Supplementary file 1 — Additional file 1: Figure S1. (A, B) The expression of macrophage phenotype genes and Smads in BMDMs with/without TGFβ1 treatment. BMDMs from naïve mice were treated with/without 10ng/ml of TGF-β1 for 96 h. Cells were collected for qRT-PCR analysis. (A) The relative mRNA expression levels of iNOS, Arg-1, Emr1 (F4/80) in control and TGF-β1 treated BMDMs. (B) Relative mRNA expression levels of Smad-1, Smad-2, and Smad-3 in control and TGFβ1-treated BMDMs. Mean ± SD, n = 3. **p<0.01, ***p < 0.001, Mann-Whitney test. (C, D) The expression of iNOS (C) and Arg-1 (D) in RPE/choroidal tissues from normal (Con) and day 5 and day 10 subretinal fibrosis mice. Mean ± SD, n = 6 eyes. **p< 0.01, Kruskal-Wallis with Dunn’s multiple comparisons test. Table S1. The list of differentially expressed genes (DEGs) in RPE-choroid from normal and subretinal fibrosis mice. Table S2. The list of significantly enriched Gene Ontology (GO) terms. [file 12974_2022_2433_MOESM1_ESM.docx]

**
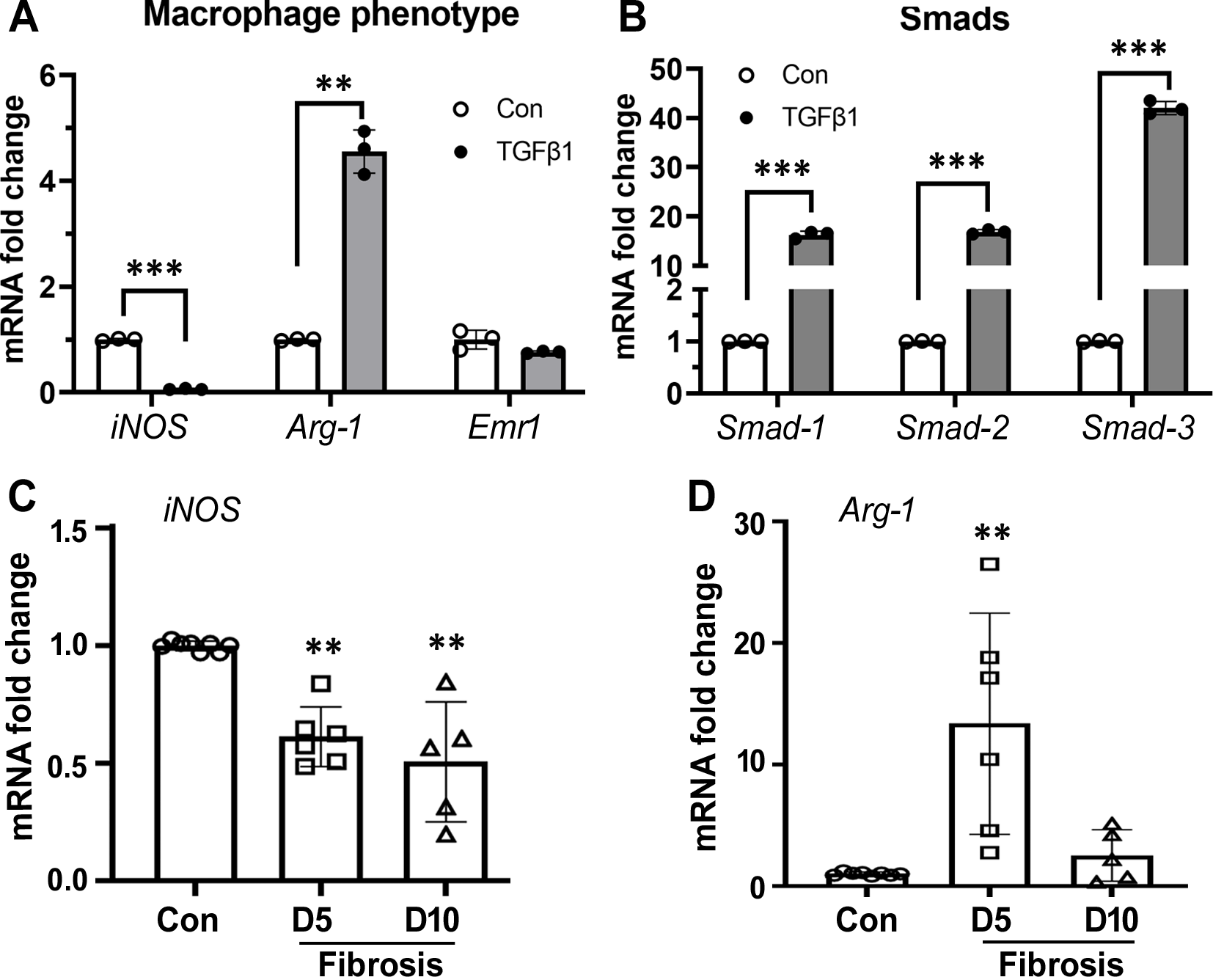
**

**Figure S1.** (A, B) The expression of macrophage phenotype genes and Smads in BMDMs with/without TGFβ1 treatment. BMDMs from naïve mice were treated with/without 10ng/ml of TGF-β1 for 96 h. Cells were collected for qRT-PCR analysis. **(A)** The relative mRNA expression levels of *iNOS, Arg-1, Emr1* (F4/80) in control and TGF-β1 treated BMDMs. **(B)** Relative mRNA expression levels of *Smad-1, Smad-2,* and *Smad-3* in control and TGFβ1-treated BMDMs. Mean ± SD, n = 3. ***p*<0.01, ****p* < 0.001, Mann-Whitney test. (C, D) The expression of iNOS (C) and Arg-1 (D) in RPE/choroidal tissues from normal (Con) and day 5 and day 10 subretinal fibrosis mice. Mean ± SD, n = 6 eyes. ***p*< 0.01, Kruskal-Wallis with Dunn’s multiple comparisons test.

**Table S1.** The list of differentially expressed genes (DEGs) in RPE-choroid from normal and subretinal fibrosis mice.

| **Symbol** | **Cont-1** | **Cont-2** | **Cont-3** | **SRF-1** | **SRF-2** | **SRF-3** | **Cont**  **mean** | **SRF**  **mean** | **log2(fc)** | **P Value** | **FDR** |
| --- | --- | --- | --- | --- | --- | --- | --- | --- | --- | --- | --- |
| Gm2000 | 1.68 | 1.14 | 1.27 | 17.86 | 8.51 | 8.65 | 1.36 | 11.67 | 3.10 | 1.37E-08 | 1.23E-05 |
| Stra6l | 0.1 | 0.15 | 0.1 | 0.33 | 1.06 | 0.95 | 0.12 | 0.78 | 2.74 | 0.000093 | 0.012717 |
| Mmp12 | 7.33 | 10.16 | 12.17 | 56.54 | 53.99 | 62.82 | 9.89 | 57.78 | 2.55 | 2.88E-30 | 4.41E-26 |
| Aard | 0.49 | 0.47 | 0.4 | 3.41 | 2.03 | 1.88 | 0.45 | 2.44 | 2.43 | 1.23E-05 | 0.002769 |
| Madcam1 | 0.5 | 0.44 | 0.81 | 2.65 | 3.1 | 2.8 | 0.58 | 2.85 | 2.29 | 1.73E-05 | 0.003498 |
| Gm49339 | 2.13 | 2.97 | 6.11 | 6.5 | 19.94 | 22.26 | 3.74 | 16.23 | 2.12 | 3.46E-05 | 0.005949 |
| Atp6v0d2 | 2.05 | 3.92 | 3.7 | 9.57 | 14.19 | 13.83 | 3.22 | 12.53 | 1.96 | 7.81E-10 | 9.96E-07 |
| Il7r | 0.39 | 0.51 | 0.32 | 1.65 | 1.59 | 1.37 | 0.41 | 1.54 | 1.92 | 1.8E-06 | 0.00069 |
| Gm13889 | 2.6 | 1.92 | 1.62 | 8.99 | 8.16 | 4.2 | 2.05 | 7.12 | 1.80 | 1.26E-05 | 0.002787 |
| Aplnr | 1.21 | 1.46 | 1.95 | 4.88 | 6.97 | 3.92 | 1.54 | 5.26 | 1.77 | 9.81E-08 | 6.63E-05 |
| Rfxap | 2.2 | 1.93 | 1.46 | 8.78 | 6.31 | 3.95 | 1.86 | 6.35 | 1.77 | 2.03E-05 | 0.003989 |
| Chil1 | 299.83 | 386.73 | 670.23 | 1378.1 | 1534.2 | 1540.72 | 452.26 | 1484.33 | 1.71 | 3.14E-09 | 3.44E-06 |
| Jund | 13.33 | 16.47 | 13.14 | 56.79 | 49.73 | 30.89 | 14.31 | 45.80 | 1.68 | 1.45E-10 | 2.22E-07 |
| Smarcd3 | 9.42 | 7.4 | 5.09 | 24.94 | 24.57 | 20.48 | 7.30 | 23.33 | 1.68 | 7.02E-11 | 1.25E-07 |
| Dexi | 11.61 | 4.95 | 4.77 | 24.72 | 21.65 | 21.48 | 7.11 | 22.62 | 1.67 | 1.55E-06 | 0.000616 |
| Ctss | 125 | 127.91 | 138.98 | 334.25 | 512.17 | 394.53 | 130.63 | 413.65 | 1.66 | 5.83E-18 | 2.97E-14 |
| Mpnd | 25.81 | 13.85 | 13.46 | 54.52 | 54.23 | 49.97 | 17.71 | 52.91 | 1.58 | 2.31E-09 | 2.72E-06 |
| Lyz2 | 554.67 | 744.44 | 745.88 | 1598 | 2570.6 | 1790.36 | 681.66 | 1986.33 | 1.54 | 2.25E-11 | 6.89E-08 |
| Bcan | 1.62 | 1.02 | 0.89 | 4.06 | 3.74 | 2.46 | 1.18 | 3.42 | 1.54 | 1.23E-05 | 0.002769 |
| Tlr13 | 1.42 | 1.38 | 1.48 | 2.59 | 5.14 | 4.38 | 1.43 | 4.04 | 1.50 | 4.64E-06 | 0.00134 |
| Cited2 | 22.59 | 11.17 | 11.52 | 44.34 | 37.92 | 45.66 | 15.09 | 42.64 | 1.50 | 2.05E-07 | 0.000121 |
| Clec7a | 7.86 | 7.81 | 8.39 | 17.51 | 28.08 | 22.28 | 8.02 | 22.62 | 1.50 | 2.91E-11 | 7.43E-08 |
| Prrx2 | 5.62 | 4.8 | 4.42 | 15.53 | 15.39 | 10.81 | 4.95 | 13.91 | 1.49 | 1.83E-10 | 2.54E-07 |
| Cop1 | 8.98 | 5.35 | 5.96 | 19.8 | 17.59 | 19.21 | 6.76 | 18.87 | 1.48 | 0.000447 | 0.046923 |
| Lat2 | 2.89 | 3.72 | 5.05 | 8.66 | 14.09 | 8.93 | 3.89 | 10.56 | 1.44 | 3.66E-05 | 0.006152 |
| Srrd | 6.45 | 3.76 | 2.66 | 13.97 | 10.86 | 9.9 | 4.29 | 11.58 | 1.43 | 0.000148 | 0.018829 |
| Ube2s | 21.83 | 16.21 | 12.76 | 56.77 | 47.62 | 31.3 | 16.93 | 45.23 | 1.42 | 2.2E-06 | 0.000759 |
| Igkc | 29.01 | 17.41 | 12.88 | 48.96 | 60.86 | 48.16 | 19.77 | 52.66 | 1.41 | 0.000008 | 0.001943 |
| Adam8 | 0.83 | 0.84 | 0.89 | 1.73 | 2.8 | 2.22 | 0.85 | 2.25 | 1.40 | 0.000101 | 0.013669 |
| Cebpb | 6.56 | 11.26 | 9.5 | 27.03 | 28.84 | 15.42 | 9.11 | 23.76 | 1.38 | 2.12E-05 | 0.004101 |
| Tpsb2 | 3.86 | 4.77 | 6.65 | 13.56 | 12.8 | 13.41 | 5.09 | 13.26 | 1.38 | 5.02E-06 | 0.001424 |
| Metrnl | 15.22 | 10.09 | 12.47 | 33.69 | 36.08 | 27.31 | 12.59 | 32.36 | 1.36 | 3.55E-11 | 7.77E-08 |
| Itgax | 0.85 | 1.09 | 1.13 | 2.94 | 2.04 | 2.9 | 1.02 | 2.63 | 1.36 | 3.08E-05 | 0.005474 |
| Glipr1 | 4.67 | 5.29 | 6.41 | 12.4 | 16.88 | 12.24 | 5.46 | 13.84 | 1.34 | 2.49E-06 | 0.000764 |
| Cma1 | 2.18 | 3.7 | 4.01 | 7.59 | 9.98 | 7.3 | 3.30 | 8.29 | 1.33 | 0.00037 | 0.03989 |
| Timp2 | 135.4 | 116.68 | 135.93 | 308.49 | 334.45 | 330.49 | 129.34 | 324.48 | 1.33 | 1.45E-20 | 1.11E-16 |
| Ino80b | 6.05 | 4.1 | 3.98 | 15.04 | 12.27 | 8.05 | 4.71 | 11.79 | 1.32 | 0.000276 | 0.031494 |
| Hkdc1 | 3.31 | 1.99 | 2.72 | 8.06 | 5.74 | 6.23 | 2.67 | 6.68 | 1.32 | 1.86E-06 | 0.00069 |
| Cyp26a1 | 18.91 | 16.35 | 35.57 | 40.24 | 68.43 | 66.65 | 23.61 | 58.44 | 1.31 | 0.000139 | 0.017971 |
| Itgb2 | 5.41 | 5.93 | 6.15 | 10.16 | 16.97 | 15.19 | 5.83 | 14.11 | 1.27 | 9.96E-08 | 6.63E-05 |
| Cd22 | 0.41 | 0.44 | 0.77 | 1.14 | 1.5 | 1.27 | 0.54 | 1.30 | 1.27 | 8.18E-05 | 0.011492 |
| Ppp1r37 | 5.79 | 4.36 | 3.33 | 11.86 | 11.7 | 8.96 | 4.49 | 10.84 | 1.27 | 6.02E-07 | 0.00028 |
| Scn1b | 16.71 | 14.14 | 10.43 | 40.99 | 35.93 | 21.19 | 13.76 | 32.70 | 1.25 | 2.78E-05 | 0.005065 |
| Fabp5 | 20.52 | 25.1 | 18.13 | 55.33 | 62.44 | 32.25 | 21.25 | 50.01 | 1.23 | 2.02E-05 | 0.003989 |
| Pirb | 2.08 | 2 | 1.63 | 2.93 | 5.53 | 4.94 | 1.90 | 4.47 | 1.23 | 0.000121 | 0.015804 |
| Vav1 | 1.07 | 1.23 | 1.41 | 2.28 | 3.27 | 2.97 | 1.24 | 2.84 | 1.20 | 0.000246 | 0.029182 |
| Btbd2 | 22.5 | 9.01 | 9.24 | 29.09 | 29.83 | 34.21 | 13.58 | 31.04 | 1.19 | 5.65E-05 | 0.008244 |
| Trem2 | 21.57 | 31.19 | 24.65 | 60.29 | 71.02 | 44.51 | 25.80 | 58.61 | 1.18 | 2.58E-06 | 0.000773 |
| Inpp5a | 7.9 | 5.91 | 3.82 | 15.26 | 14.16 | 9.57 | 5.88 | 13.00 | 1.15 | 0.000257 | 0.02999 |
| Cmas | 18.4 | 11.38 | 15.46 | 35.81 | 31.01 | 32.22 | 15.08 | 33.01 | 1.13 | 3.17E-07 | 0.000173 |
| Bcl2a1b | 14.17 | 14.69 | 15.53 | 34.97 | 35.66 | 26.53 | 14.80 | 32.39 | 1.13 | 3.4E-07 | 0.000173 |
| Cd68 | 54.76 | 70.54 | 61.04 | 127.2 | 157.81 | 113.45 | 62.11 | 132.82 | 1.10 | 3.04E-08 | 2.59E-05 |
| Ankrd13a | 5.08 | 4.27 | 4.08 | 10.62 | 10.21 | 7.86 | 4.48 | 9.56 | 1.10 | 1.57E-06 | 0.000616 |
| Smad1 | 7.18 | 7.86 | 7.86 | 19.52 | 15.21 | 13.82 | 7.63 | 16.18 | 1.08 | 1.75E-07 | 0.000107 |
| Ms4a7 | 43.29 | 48.51 | 45.54 | 94.44 | 109.92 | 85.85 | 45.78 | 96.74 | 1.08 | 7.33E-11 | 1.25E-07 |
| Ccdc71l | 1.21 | 1.42 | 0.95 | 2.49 | 2.49 | 2.53 | 1.19 | 2.50 | 1.07 | 0.00022 | 0.026561 |
| Rnf126 | 4.28 | 2.84 | 3.03 | 7.45 | 7.72 | 6.1 | 3.38 | 7.09 | 1.07 | 0.000245 | 0.029182 |
| Cd5l | 2.69 | 2.57 | 2.52 | 4.3 | 6.52 | 5.38 | 2.59 | 5.40 | 1.06 | 0.000315 | 0.034898 |
| Vac14 | 6.49 | 5.59 | 5.14 | 11.41 | 12.22 | 12.2 | 5.74 | 11.94 | 1.06 | 7.56E-09 | 7.71E-06 |
| Lilr4b | 6.28 | 6.56 | 7.49 | 11.42 | 16.77 | 14.06 | 6.78 | 14.08 | 1.06 | 8.89E-05 | 0.01226 |
| Cd84 | 7.65 | 6.88 | 7.42 | 12.27 | 16.99 | 16.27 | 7.32 | 15.18 | 1.05 | 1.89E-06 | 0.00069 |
| Tle1 | 7.85 | 4.09 | 4.83 | 10.26 | 12.77 | 11.18 | 5.59 | 11.40 | 1.03 | 3.96E-05 | 0.006519 |
| Zfp423 | 9.86 | 8.49 | 5.14 | 17.94 | 17.79 | 11.77 | 7.83 | 15.83 | 1.02 | 6.98E-06 | 0.001751 |
| C3ar1 | 6.18 | 6.49 | 6.29 | 10.63 | 13.75 | 13.9 | 6.32 | 12.76 | 1.01 | 9.67E-08 | 6.63E-05 |
| Slc36a2 | 3.49 | 5.06 | 4.92 | 8.76 | 10.77 | 7.48 | 4.49 | 9.00 | 1.00 | 0.000275 | 0.031494 |
| Spi1 | 6.83 | 6.9 | 8.15 | 15.93 | 13.99 | 13.48 | 7.29 | 14.47 | 0.99 | 2.04E-06 | 0.000725 |
| Csf2ra | 8.18 | 7.82 | 6.27 | 15.22 | 15.95 | 12.54 | 7.42 | 14.57 | 0.97 | 6.63E-06 | 0.001692 |
| Rnf128 | 8.15 | 8.6 | 7.23 | 12.58 | 18.98 | 15.35 | 7.99 | 15.64 | 0.97 | 1.57E-05 | 0.003299 |
| Cbx4 | 3.22 | 2 | 2.17 | 4.52 | 4.79 | 5.13 | 2.46 | 4.81 | 0.97 | 0.000109 | 0.014502 |
| Itgb5 | 39.05 | 38.2 | 36.19 | 68.7 | 79.44 | 73.22 | 37.81 | 73.79 | 0.96 | 1.21E-15 | 4.64E-12 |
| Mtch1 | 126.31 | 105.75 | 85.84 | 243.65 | 207.62 | 168.9 | 105.97 | 206.72 | 0.96 | 1.37E-06 | 0.000567 |
| C1qb | 107.55 | 143.49 | 127.51 | 240.83 | 287.65 | 205.01 | 126.18 | 244.50 | 0.95 | 2.39E-06 | 0.000759 |
| Rnf103 | 18.42 | 9.73 | 10.69 | 24.85 | 21.36 | 27.59 | 12.95 | 24.60 | 0.93 | 0.000336 | 0.036745 |
| Acp5 | 9.3 | 8.69 | 9.34 | 18.71 | 17.48 | 15.44 | 9.11 | 17.21 | 0.92 | 6.21E-06 | 0.001612 |
| Sox4 | 4.76 | 4.99 | 5.64 | 11.24 | 11.12 | 6.71 | 5.13 | 9.69 | 0.92 | 0.000417 | 0.044592 |
| Laptm5 | 27.67 | 32.45 | 34.08 | 50.78 | 69.2 | 56.43 | 31.40 | 58.80 | 0.91 | 3.01E-05 | 0.00542 |
| Hvcn1 | 9.42 | 10.24 | 10.98 | 16.67 | 21.29 | 18.7 | 10.21 | 18.89 | 0.89 | 2.38E-06 | 0.000759 |
| Abhd17c | 7.48 | 6.24 | 5.04 | 11.27 | 12.76 | 10.47 | 6.25 | 11.50 | 0.88 | 0.000144 | 0.018499 |
| Fhl2 | 11.51 | 9.38 | 11.71 | 19.43 | 23.87 | 16.48 | 10.87 | 19.93 | 0.87 | 0.000174 | 0.021456 |
| Cd53 | 16.79 | 18.96 | 19.89 | 29.4 | 38.71 | 33.79 | 18.55 | 33.97 | 0.87 | 7.42E-06 | 0.001832 |
| Tmem65 | 7.63 | 7.09 | 6.82 | 11.71 | 13.28 | 14.4 | 7.18 | 13.13 | 0.87 | 7.23E-07 | 0.000316 |
| E2f6 | 12.82 | 10.03 | 10.68 | 19.66 | 19.86 | 21.63 | 11.18 | 20.38 | 0.87 | 7.09E-07 | 0.000316 |
| C1qc | 89.12 | 122.56 | 125.36 | 192.38 | 236.84 | 184.52 | 112.35 | 204.58 | 0.86 | 4.24E-05 | 0.006695 |
| Fcrls | 6.24 | 8.2 | 8.67 | 12.14 | 14.5 | 15.39 | 7.70 | 14.01 | 0.86 | 0.000456 | 0.047517 |
| Dok7 | 2.54 | 2.49 | 2.36 | 4.33 | 4.5 | 4.58 | 2.46 | 4.47 | 0.86 | 0.000327 | 0.035985 |
| Cybb | 8.74 | 8.88 | 9.86 | 15.33 | 17.49 | 16.95 | 9.16 | 16.59 | 0.86 | 9.39E-08 | 6.63E-05 |
| Tyrobp | 103.15 | 114 | 108.23 | 192.76 | 234.98 | 159.29 | 108.46 | 195.68 | 0.85 | 1.56E-05 | 0.003299 |
| Lrig3 | 4.61 | 5.59 | 4.55 | 10.51 | 8.53 | 7.52 | 4.92 | 8.85 | 0.85 | 0.000173 | 0.021456 |
| Wfdc17 | 67.64 | 91.28 | 83.02 | 138.1 | 165.03 | 130.88 | 80.65 | 144.67 | 0.84 | 3.77E-05 | 0.006269 |
| Adgra3 | 4.42 | 5.23 | 5.13 | 8.12 | 8.23 | 9.64 | 4.93 | 8.66 | 0.81 | 4.06E-05 | 0.006597 |
| Fcer1g | 90.6 | 97.79 | 93.35 | 172.29 | 188.51 | 133.14 | 93.91 | 164.65 | 0.81 | 1.66E-05 | 0.003444 |
| Ly86 | 10.13 | 10.18 | 10.89 | 17.52 | 19.45 | 17.72 | 10.40 | 18.23 | 0.81 | 7.51E-05 | 0.010651 |
| Zdhhc2 | 11.46 | 9.04 | 7.02 | 17.25 | 15.26 | 15.48 | 9.17 | 16.00 | 0.80 | 0.000431 | 0.045497 |
| Cd300a | 2.44 | 2.95 | 2.44 | 4.93 | 4.61 | 4.03 | 2.61 | 4.52 | 0.79 | 0.000471 | 0.048362 |
| Rftn1 | 9.14 | 10.41 | 12.08 | 16.97 | 18.16 | 18.74 | 10.54 | 17.96 | 0.77 | 0.000119 | 0.015683 |
| Serpine2 | 343.81 | 402.66 | 486.4 | 593.66 | 774.19 | 703.28 | 410.96 | 690.38 | 0.75 | 0.000297 | 0.033444 |
| Sidt2 | 16.08 | 14.74 | 12.95 | 25.45 | 26.14 | 21.49 | 14.59 | 24.36 | 0.74 | 1.54E-05 | 0.003299 |
| Tmem165 | 19.31 | 14.71 | 17.44 | 28.5 | 27.11 | 29.93 | 17.15 | 28.51 | 0.73 | 5.95E-05 | 0.008597 |
| Nt5dc2 | 88.85 | 90.22 | 77.18 | 166.89 | 141.98 | 114.01 | 85.42 | 140.96 | 0.72 | 5.02E-05 | 0.007606 |
| Adora1 | 11.7 | 10.97 | 14.83 | 17.17 | 21.72 | 20.85 | 12.50 | 19.91 | 0.67 | 0.0002 | 0.024249 |
| Spp1 | 442.63 | 309.49 | 357.64 | 582.12 | 553.36 | 584.62 | 369.92 | 573.37 | 0.63 | 0.000273 | 0.031494 |
| Mtmr3 | 7.78 | 7.22 | 7.47 | 11.52 | 11.88 | 11.16 | 7.49 | 11.52 | 0.62 | 1.74E-05 | 0.003498 |
| Mfge8 | 123.5 | 112.76 | 112.53 | 169.19 | 171.9 | 192.25 | 116.26 | 177.78 | 0.61 | 5.59E-06 | 0.001477 |
| Rragc | 15.6 | 12.96 | 12.71 | 21.79 | 21.86 | 19.34 | 13.76 | 21.00 | 0.61 | 0.000465 | 0.048136 |
| Elovl1 | 192.02 | 192.02 | 181.84 | 144.45 | 127.93 | 121.89 | 188.63 | 131.42 | -0.52 | 0.000014 | 0.003052 |
| Lratd2 | 18.02 | 17.65 | 17.81 | 12.09 | 12.94 | 12.05 | 17.83 | 12.36 | -0.53 | 1.34E-06 | 0.000567 |
| Col4a3 | 35.34 | 33.51 | 37.73 | 25.28 | 23.43 | 25.15 | 35.53 | 24.62 | -0.53 | 2.3E-06 | 0.000759 |
| F3 | 103.42 | 114.42 | 112.1 | 83.96 | 74.79 | 67.06 | 109.98 | 75.27 | -0.55 | 4.09E-05 | 0.006597 |
| Slco1a4 | 123.89 | 120.97 | 142.84 | 83.11 | 76.5 | 105.37 | 129.23 | 88.33 | -0.55 | 5.64E-05 | 0.008244 |
| Rgr | 7583.58 | 8745.13 | 7800.95 | 6273.1 | 4961.2 | 5154.25 | 8043.22 | 5462.86 | -0.56 | 5.6E-07 | 0.000276 |
| Slc16a1 | 173.89 | 184.52 | 184.62 | 118.8 | 107.74 | 139.06 | 181.01 | 121.87 | -0.57 | 2.68E-05 | 0.004946 |
| Tshr | 70.03 | 74.05 | 74.17 | 52.8 | 44.22 | 49.77 | 72.75 | 48.93 | -0.57 | 1.11E-05 | 0.002622 |
| Chac2 | 28.65 | 25.78 | 23.89 | 17.45 | 17.47 | 17.36 | 26.11 | 17.43 | -0.58 | 4.72E-05 | 0.007304 |
| Tob1 | 51.78 | 52.52 | 47.97 | 28.41 | 35.68 | 37.47 | 50.76 | 33.85 | -0.58 | 7.13E-05 | 0.010196 |
| Gtf2a1 | 40.05 | 38.43 | 44.87 | 23.87 | 26.1 | 31.09 | 41.12 | 27.02 | -0.61 | 0.000043 | 0.006713 |
| Coq10b | 26.04 | 30.61 | 33.41 | 17.97 | 18.16 | 22.61 | 30.02 | 19.58 | -0.62 | 0.000429 | 0.045497 |
| Slc6a13 | 199.27 | 223.06 | 195.33 | 145.66 | 128.37 | 126.78 | 205.89 | 133.60 | -0.62 | 3.36E-07 | 0.000173 |
| Sult1c1 | 121.21 | 116.77 | 100.64 | 80.33 | 67.41 | 71.02 | 112.87 | 72.92 | -0.63 | 2.4E-06 | 0.000759 |
| Lrat | 319.67 | 365.59 | 368.17 | 232.49 | 191.24 | 229.23 | 351.14 | 217.65 | -0.69 | 5.9E-07 | 0.00028 |
| Mogat1 | 71.58 | 71.66 | 60.29 | 51.38 | 39.61 | 34.45 | 67.84 | 41.81 | -0.70 | 0.000481 | 0.049074 |
| Upk3b | 13.76 | 14.4 | 10.6 | 8.93 | 7.17 | 7.74 | 12.92 | 7.95 | -0.70 | 0.000106 | 0.014235 |
| Dusp6 | 85.27 | 111.84 | 117.29 | 71.08 | 66.3 | 54.33 | 104.80 | 63.90 | -0.71 | 4.24E-05 | 0.006695 |
| Htr3a | 32.92 | 32.85 | 31.15 | 22.92 | 14.6 | 20.95 | 32.31 | 19.49 | -0.73 | 0.00016 | 0.020292 |
| Rd3l | 227.57 | 226.84 | 170.69 | 150.42 | 120 | 105.59 | 208.37 | 125.34 | -0.73 | 5.55E-05 | 0.008244 |
| Rrh | 378.81 | 339.2 | 340.82 | 232.15 | 183.18 | 220.39 | 352.94 | 211.91 | -0.74 | 9.05E-08 | 6.63E-05 |
| Ermap | 14.74 | 14.15 | 15.26 | 9.87 | 7.25 | 8.96 | 14.72 | 8.69 | -0.76 | 3.34E-06 | 0.000983 |
| Fam13a | 42.75 | 47.93 | 37.84 | 28.39 | 29.06 | 18.25 | 42.84 | 25.23 | -0.76 | 3.34E-05 | 0.00587 |
| Adora2b | 61.04 | 64.79 | 61.74 | 39.78 | 37.26 | 31.78 | 62.52 | 36.27 | -0.79 | 8.13E-09 | 7.78E-06 |
| Spry2 | 49.94 | 74.25 | 63.43 | 35.22 | 34.01 | 36.08 | 62.54 | 35.10 | -0.83 | 2.43E-06 | 0.000759 |
| Krt23 | 30.75 | 36.53 | 30.16 | 22.62 | 16.57 | 15.22 | 32.48 | 18.14 | -0.84 | 2.23E-05 | 0.00426 |
| Map6d1 | 10.66 | 13.64 | 11.88 | 7.09 | 5.37 | 7.23 | 12.06 | 6.56 | -0.88 | 8.56E-06 | 0.002047 |
| Gzmm | 38.3 | 47.59 | 37.98 | 25 | 19.93 | 20.08 | 41.29 | 21.67 | -0.93 | 2.34E-07 | 0.000133 |
| Sik1 | 1.6 | 4.48 | 3.95 | 0.98 | 1.16 | 1.1 | 3.34 | 1.08 | -1.63 | 0.000023 | 0.004346 |
| Gpr139 | 1.4 | 1.63 | 2.23 | 0.58 | 0.28 | 0.42 | 1.75 | 0.43 | -2.04 | 1.25E-07 | 0.00008 |
| Arc | 1.03 | 2.1 | 1.49 | 0.36 | 0.19 | 0.44 | 1.54 | 0.33 | -2.22 | 1.18E-05 | 0.002729 |
| Gm20075 | 1.44 | 1.3 | 1.28 | 0.3 | 0.16 | 0.4 | 1.34 | 0.29 | -2.22 | 3.61E-05 | 0.00614 |
| Egr3 | 0.45 | 2.32 | 0.99 | 0.19 | 0.2 | 0.32 | 1.25 | 0.24 | -2.40 | 0.000182 | 0.022324 |
| Nr4a1 | 31.2 | 143.3 | 115.05 | 11.38 | 9.66 | 19.83 | 96.52 | 13.62 | -2.82 | 2.58E-05 | 0.004811 |
| Crygb | 0.58 | 11.42 | 4.34 | 0.35 | 0.13 | 0.45 | 5.45 | 0.31 | -4.14 | 3.42E-05 | 0.005949 |

**Table S2.** The list of significantly enriched Gene Ontology (GO) terms.

| Class | ID | Description | p value | q-value |
| --- | --- | --- | --- | --- |
| Biological Process | GO:0006954 | Inflammatory response | 4.54E-09 | 1.46E-05 |
| Biological Process | GO:0048514 | Blood vessel morphogenesis | 2.34E-07 | 0.000243 |
| Biological Process | GO:0002376 | Immune system process | 2.54E-07 | 0.000243 |
| Biological Process | GO:0007229 | Integrin-mediated signaling pathway | 3.05E-07 | 0.000243 |
| Biological Process | GO:0006952 | Defense response | 4.42E-07 | 0.000243 |
| Biological Process | GO:0002684 | Positive regulation of immune system process | 5.96E-07 | 0.000243 |
| Biological Process | GO:0007166 | Cell surface receptor signaling pathway | 5.97E-07 | 0.000243 |
| Biological Process | GO:0048518 | Positive regulation of biological process | 6.06E-07 | 0.000243 |
| Biological Process | GO:0002275 | Myeloid cell activation involved in immune response | 7.5E-07 | 0.00025 |
| Biological Process | GO:0001525 | Angiogenesis | 8.02E-07 | 0.00025 |
| Biological Process | GO:0002682 | Regulation of immune system process | 9.07E-07 | 0.00025 |
| Biological Process | GO:0009605 | Response to external stimulus | 9.36E-07 | 0.00025 |
| Biological Process | GO:0010033 | Response to organic substance | 1.1E-06 | 0.000271 |
| Biological Process | GO:0072358 | Cardiovascular system development | 1.51E-06 | 0.000323 |
| Biological Process | GO:0072359 | Circulatory system development | 1.51E-06 | 0.000323 |
| Biological Process | GO:0045321 | Leukocyte activation | 1.73E-06 | 0.000331 |
| Biological Process | GO:0001568 | Blood vessel development | 1.81E-06 | 0.000331 |
| Biological Process | GO:0070887 | Cellular response to chemical stimulus | 1.86E-06 | 0.000331 |
| Biological Process | GO:0050776 | Regulation of immune response | 2.24E-06 | 0.00034 |
| Biological Process | GO:0051239 | Regulation of multicellular organismal process | 2.28E-06 | 0.00034 |
| Biological Process | GO:0002443 | Leukocyte mediated immunity | 2.29E-06 | 0.00034 |
| Biological Process | GO:0006955 | Immune response | 2.34E-06 | 0.00034 |
| Biological Process | GO:0001775 | Cell activation | 2.43E-06 | 0.00034 |
| Biological Process | GO:0001944 | Vasculature development | 2.77E-06 | 0.000355 |
| Biological Process | GO:0048646 | Anatomical structure formation involved in morphogenesis | 2.86E-06 | 0.000355 |
| Biological Process | GO:0071310 | Cellular response to organic substance | 2.87E-06 | 0.000355 |
| Biological Process | GO:0044707 | Single-multicellular organism process | 3.07E-06 | 0.000365 |
| Biological Process | GO:0002274 | Myeloid leukocyte activation | 6.33E-06 | 0.000725 |
| Biological Process | GO:0002252 | Immune effector process | 6.67E-06 | 0.000738 |
| Biological Process | GO:0031347 | Regulation of defense response | 7.27E-06 | 0.000777 |
| Biological Process | GO:0002521 | Leukocyte differentiation | 9.34E-06 | 0.000967 |
| Biological Process | GO:0002573 | Myeloid leukocyte differentiation | 9.7E-06 | 0.000973 |
| Biological Process | GO:0043303 | Mast cell degranulation | 1.11E-05 | 0.001084 |
| Biological Process | GO:0002279 | Mast cell activation involved in immune response | 1.24E-05 | 0.001138 |
| Biological Process | GO:0002448 | Mast cell mediated immunity | 1.24E-05 | 0.001138 |
| Biological Process | GO:0050778 | Positive regulation of immune response | 1.32E-05 | 0.001175 |
| Biological Process | GO:0045766 | Positive regulation of angiogenesis | 1.36E-05 | 0.001177 |
| Biological Process | GO:0001816 | Cytokine production | 1.46E-05 | 0.001234 |
| Biological Process | GO:0032879 | Regulation of localization | 1.86E-05 | 0.001529 |
| Biological Process | GO:0045765 | Regulation of angiogenesis | 1.98E-05 | 0.00159 |
| Biological Process | GO:0051049 | Regulation of transport | 2.13E-05 | 0.001669 |
| Biological Process | GO:0002444 | Myeloid leukocyte mediated immunity | 2.48E-05 | 0.001898 |
| Biological Process | GO:1904018 | Positive regulation of vasculature development | 2.71E-05 | 0.002022 |
| Biological Process | GO:0002886 | Regulation of myeloid leukocyte mediated immunity | 3.22E-05 | 0.002347 |
| Biological Process | GO:1901342 | Regulation of vasculature development | 3.87E-05 | 0.002761 |
| Biological Process | GO:0048584 | Positive regulation of response to stimulus | 4.26E-05 | 0.002795 |
| Biological Process | GO:0016337 | Single organismal cell-cell adhesion | 4.31E-05 | 0.002795 |
| Biological Process | GO:0043269 | Regulation of ion transport | 4.31E-05 | 0.002795 |
| Biological Process | GO:0002683 | Negative regulation of immune system process | 4.34E-05 | 0.002795 |
| Biological Process | GO:0050900 | Leukocyte migration | 4.42E-05 | 0.002795 |
| Biological Process | GO:0043304 | Regulation of mast cell degranulation | 4.44E-05 | 0.002795 |
| Biological Process | GO:0051240 | Positive regulation of multicellular organismal process | 4.77E-05 | 0.002945 |
| Biological Process | GO:0033006 | Regulation of mast cell activation involved in immune response | 5.05E-05 | 0.003059 |
| Biological Process | GO:0045576 | Mast cell activation | 5.24E-05 | 0.003114 |
| Biological Process | GO:0051094 | Positive regulation of developmental process | 5.46E-05 | 0.003186 |
| Biological Process | GO:0042592 | Homeostatic process | 5.71E-05 | 0.003272 |
| Biological Process | GO:0009653 | Anatomical structure morphogenesis | 5.95E-05 | 0.00335 |
| Biological Process | GO:0043299 | Leukocyte degranulation | 6.56E-05 | 0.00363 |
| Biological Process | GO:0006950 | Response to stress | 7.2E-05 | 0.003916 |
| Cellular Component | GO:0009986 | Cell surface | 1.22E-05 | 0.004117 |
| Biological Process | GO:0031349 | Positive regulation of defense response | 8.24E-05 | 0.004406 |
| Biological Process | GO:0048583 | Regulation of response to stimulus | 8.92E-05 | 0.004693 |
| Biological Process | GO:0065007 | Biological regulation | 9.36E-05 | 0.004845 |
| Biological Process | GO:0032418 | Lysosome localization | 9.96E-05 | 0.005073 |
| Biological Process | GO:0042107 | Cytokine metabolic process | 0.000108 | 0.005266 |
| Biological Process | GO:0033089 | Positive regulation of T cell differentiation in thymus | 0.000111 | 0.005266 |
| Biological Process | GO:1900452 | Regulation of long term synaptic depression | 0.000111 | 0.005266 |
| Biological Process | GO:2000400 | Positive regulation of thymocyte aggregation | 0.000111 | 0.005266 |
| Biological Process | GO:0097028 | Dendritic cell differentiation | 0.000112 | 0.005266 |
| Biological Process | GO:0002703 | Regulation of leukocyte mediated immunity | 0.000121 | 0.005508 |
| Biological Process | GO:0002282 | Microglial cell activation involved in immune response | 0.000122 | 0.005508 |
| Biological Process | GO:0034769 | Basement membrane disassembly | 0.000122 | 0.005508 |
| Biological Process | GO:0098602 | Single organism cell adhesion | 0.000127 | 0.00563 |
| Biological Process | GO:0050896 | Response to stimulus | 0.000128 | 0.00563 |
| Biological Process | GO:0046649 | Lymphocyte activation | 0.000146 | 0.006324 |
| Biological Process | GO:0048513 | Animal organ development | 0.000148 | 0.006324 |
| Biological Process | GO:0048731 | System development | 0.000153 | 0.006446 |
| Biological Process | GO:0043270 | Positive regulation of ion transport | 0.000166 | 0.006908 |
| Biological Process | GO:0033003 | Regulation of mast cell activation | 0.00018 | 0.007299 |
| Biological Process | GO:0043300 | Regulation of leukocyte degranulation | 0.00018 | 0.007299 |
| Biological Process | GO:0050727 | Regulation of inflammatory response | 0.000183 | 0.007323 |
| Biological Process | GO:0002697 | Regulation of immune effector process | 0.0002 | 0.007941 |
| Biological Process | GO:0050793 | Regulation of developmental process | 0.000218 | 0.008521 |
| Biological Process | GO:0097529 | myeloid leukocyte migration | 0.000221 | 0.008532 |
| Biological Process | GO:0034097 | response to cytokine | 0.000233 | 0.008886 |
| Biological Process | GO:0002351 | serotonin production involved in inflammatory response | 0.000243 | 0.00889 |
| Biological Process | GO:0002442 | serotonin secretion involved in inflammatory response | 0.000243 | 0.00889 |
| Biological Process | GO:0051716 | cellular response to stimulus | 0.000244 | 0.00889 |
| Biological Process | GO:0032101 | regulation of response to external stimulus | 0.000244 | 0.00889 |
| Biological Process | GO:0097530 | granulocyte migration | 0.000247 | 0.00889 |
| Biological Process | GO:0048522 | positive regulation of cellular process | 0.000251 | 0.008933 |
| Biological Process | GO:0010604 | positive regulation of macromolecule metabolic process | 0.000257 | 0.009013 |
| Biological Process | GO:0008283 | cell proliferation | 0.000263 | 0.009013 |
| Biological Process | GO:0001817 | regulation of cytokine production | 0.000264 | 0.009013 |
| Biological Process | GO:0002366 | leukocyte activation involved in immune response | 0.000264 | 0.009013 |
| Biological Process | GO:0002263 | cell activation involved in immune response | 0.000285 | 0.009545 |
| Biological Process | GO:0031325 | positive regulation of cellular metabolic process | 0.000286 | 0.009545 |
| Biological Process | GO:1900271 | regulation of long-term synaptic potentiation | 0.000296 | 0.009789 |
| Biological Process | GO:0007275 | multicellular organism development | 0.000315 | 0.010321 |
| Cellular Component | GO:0000323 | lytic vacuole | 0.000109 | 0.011075 |
| Cellular Component | GO:0005764 | lysosome | 0.000109 | 0.011075 |
| Cellular Component | GO:0005773 | vacuole | 0.000152 | 0.011075 |
| Cellular Component | GO:0005768 | endosome | 0.000164 | 0.011075 |
| Biological Process | GO:0030810 | positive regulation of nucleotide biosynthetic process | 0.000371 | 0.011895 |
| Biological Process | GO:1900373 | positive regulation of purine nucleotide biosynthetic process | 0.000371 | 0.011895 |
| Biological Process | GO:0009893 | positive regulation of metabolic process | 0.000397 | 0.01243 |
| Biological Process | GO:0033227 | dsRNA transport | 0.000403 | 0.01243 |
| Biological Process | GO:0034239 | regulation of macrophage fusion | 0.000403 | 0.01243 |
| Biological Process | GO:0034241 | positive regulation of macrophage fusion | 0.000403 | 0.01243 |
| Biological Process | GO:0060291 | long-term synaptic potentiation | 0.000423 | 0.012936 |
| Biological Process | GO:0002253 | activation of immune response | 0.000438 | 0.01326 |
| Biological Process | GO:0001819 | positive regulation of cytokine production | 0.000461 | 0.013839 |
| Biological Process | GO:2000026 | regulation of multicellular organismal development | 0.000474 | 0.01408 |
| Biological Process | GO:0009611 | response to wounding | 0.000485 | 0.014278 |
| Biological Process | GO:1903555 | regulation of tumor necrosis factor superfamily cytokine production | 0.000513 | 0.014979 |
| Biological Process | GO:0019882 | antigen processing and presentation | 0.000532 | 0.015377 |
| Biological Process | GO:0044700 | single organism signaling | 0.000545 | 0.015599 |
| Biological Process | GO:0007159 | leukocyte cell-cell adhesion | 0.000549 | 0.015599 |
| Biological Process | GO:0002673 | regulation of acute inflammatory response | 0.000559 | 0.015744 |
| Biological Process | GO:0023052 | signaling | 0.000571 | 0.015821 |
| Biological Process | GO:0071706 | tumor necrosis factor superfamily cytokine production | 0.000587 | 0.015821 |
| Biological Process | GO:0042119 | neutrophil activation | 0.000601 | 0.015821 |
| Biological Process | GO:0034238 | macrophage fusion | 0.000602 | 0.015821 |
| Biological Process | GO:0043305 | negative regulation of mast cell degranulation | 0.000602 | 0.015821 |
| Biological Process | GO:0050720 | interleukin-1 beta biosynthetic process | 0.000602 | 0.015821 |
| Biological Process | GO:0050859 | negative regulation of B cell receptor signaling pathway | 0.000602 | 0.015821 |
| Biological Process | GO:2000416 | regulation of eosinophil migration | 0.000602 | 0.015821 |
| Biological Process | GO:0030316 | osteoclast differentiation | 0.000606 | 0.015821 |
| Biological Process | GO:0042127 | regulation of cell proliferation | 0.000631 | 0.016302 |
| Biological Process | GO:0000768 | syncytium formation by plasma membrane fusion | 0.000635 | 0.016302 |
| Biological Process | GO:1903034 | regulation of response to wounding | 0.000679 | 0.017266 |
| Biological Process | GO:0002250 | adaptive immune response | 0.000683 | 0.017266 |
| Biological Process | GO:0006949 | syncytium formation | 0.000718 | 0.017993 |
| Biological Process | GO:0042089 | cytokine biosynthetic process | 0.000748 | 0.018348 |
| Biological Process | GO:1990266 | neutrophil migration | 0.000748 | 0.018348 |
| Biological Process | GO:0008152 | metabolic process | 0.000749 | 0.018348 |
| Biological Process | GO:0002699 | positive regulation of immune effector process | 0.000813 | 0.019775 |
| Biological Process | GO:0030099 | myeloid cell differentiation | 0.000827 | 0.01994 |
| Biological Process | GO:0007155 | cell adhesion | 0.000833 | 0.01994 |
| Biological Process | GO:0060087 | relaxation of vascular smooth muscle | 0.000839 | 0.01994 |
| Biological Process | GO:0007154 | cell communication | 0.000872 | 0.020584 |
| Biological Process | GO:0022610 | biological adhesion | 0.000915 | 0.021005 |
| Biological Process | GO:0033081 | regulation of T cell differentiation in thymus | 0.000922 | 0.021005 |
| Biological Process | GO:0060143 | positive regulation of syncytium formation by plasma membrane fusion | 0.000922 | 0.021005 |
| Biological Process | GO:2000398 | regulation of thymocyte aggregation | 0.000922 | 0.021005 |
| Biological Process | GO:0051179 | localization | 0.000923 | 0.021005 |
| Biological Process | GO:0008285 | negative regulation of cell proliferation | 0.000959 | 0.021672 |
| Cellular Component | GO:0044459 | plasma membrane part | 0.000389 | 0.02189 |
| Biological Process | GO:0048519 | negative regulation of biological process | 0.001027 | 0.023056 |
| Biological Process | GO:0050729 | positive regulation of inflammatory response | 0.001062 | 0.023604 |
| Biological Process | GO:0051050 | positive regulation of transport | 0.001069 | 0.023604 |
| Biological Process | GO:0051234 | establishment of localization | 0.00111 | 0.023604 |
| Biological Process | GO:0033007 | negative regulation of mast cell activation involved in immune response | 0.001114 | 0.023604 |
| Biological Process | GO:0035360 | positive regulation of peroxisome proliferator activated receptor signaling pathway | 0.001114 | 0.023604 |
| Biological Process | GO:0042222 | interleukin-1 biosynthetic process | 0.001114 | 0.023604 |
| Biological Process | GO:0060142 | regulation of syncytium formation by plasma membrane fusion | 0.001115 | 0.023604 |
| Biological Process | GO:0060292 | long term synaptic depression | 0.001115 | 0.023604 |
| Biological Process | GO:0045453 | bone resorption | 0.001125 | 0.023604 |
| Biological Process | GO:0045670 | regulation of osteoclast differentiation | 0.001125 | 0.023604 |
| Biological Process | GO:0001649 | osteoblast differentiation | 0.001147 | 0.023904 |
| Biological Process | GO:0030097 | hemopoiesis | 0.001168 | 0.02419 |
| Biological Process | GO:0002761 | regulation of myeloid leukocyte differentiation | 0.001186 | 0.024391 |
| Biological Process | GO:0002520 | immune system development | 0.001195 | 0.024429 |
| Biological Process | GO:0036230 | granulocyte activation | 0.001221 | 0.024801 |
| Mol Function | GO:0001972 | retinoic acid binding | 9.27E-05 | 0.024953 |
| Mol Function | GO:0005178 | integrin binding | 0.000112 | 0.024953 |
| Biological Process | GO:0098609 | cell-cell adhesion | 0.001298 | 0.026174 |
| Biological Process | GO:0006811 | ion transport | 0.001307 | 0.026174 |
| Biological Process | GO:0001938 | positive regulation of endothelial cell proliferation | 0.001313 | 0.026174 |
| Biological Process | GO:0023056 | positive regulation of signaling | 0.001332 | 0.026244 |
| Biological Process | GO:0018345 | protein palmitoylation | 0.001333 | 0.026244 |
| Biological Process | GO:0015850 | organic hydroxy compound transport | 0.001366 | 0.026594 |
| Biological Process | GO:0050789 | regulation of biological process | 0.001367 | 0.026594 |
| Biological Process | GO:0031323 | regulation of cellular metabolic process | 0.001398 | 0.026885 |
| Biological Process | GO:1902578 | single-organism localization | 0.001399 | 0.026885 |
| Biological Process | GO:0002887 | negative regulation of myeloid leukocyte mediated immunity | 0.001426 | 0.027077 |
| Biological Process | GO:0044557 | relaxation of smooth muscle | 0.001426 | 0.027077 |
| Biological Process | GO:0048856 | anatomical structure development | 0.001446 | 0.02729 |
| Biological Process | GO:0002685 | regulation of leukocyte migration | 0.001468 | 0.027556 |
| Biological Process | GO:0050866 | negative regulation of cell activation | 0.001508 | 0.027878 |
| Biological Process | GO:0044767 | single-organism developmental process | 0.001509 | 0.027878 |
| Biological Process | GO:0044765 | single-organism transport | 0.001512 | 0.027878 |
| Biological Process | GO:0042554 | superoxide anion generation | 0.001576 | 0.028895 |
| Biological Process | GO:0023051 | regulation of signaling | 0.001602 | 0.029211 |
| Biological Process | GO:0010647 | positive regulation of cell communication | 0.001637 | 0.029662 |
| Biological Process | GO:0006915 | apoptotic process | 0.00165 | 0.029662 |
| Biological Process | GO:0032103 | positive regulation of response to external stimulus | 0.001655 | 0.029662 |
| Biological Process | GO:0002888 | positive regulation of myeloid leukocyte mediated immunity | 0.001707 | 0.030429 |
| Biological Process | GO:0019222 | regulation of metabolic process | 0.001742 | 0.030593 |
| Biological Process | GO:0045981 | positive regulation of nucleotide metabolic process | 0.001752 | 0.030593 |
| Biological Process | GO:1900544 | positive regulation of purine nucleotide metabolic process | 0.001752 | 0.030593 |
| Biological Process | GO:0071345 | cellular response to cytokine stimulus | 0.001754 | 0.030593 |
| Biological Process | GO:0003211 | cardiac ventricle formation | 0.001775 | 0.030789 |
| Biological Process | GO:0071704 | organic substance metabolic process | 0.001801 | 0.031075 |
| Biological Process | GO:0010646 | regulation of cell communication | 0.001841 | 0.03148 |
| Biological Process | GO:0030851 | granulocyte differentiation | 0.001845 | 0.03148 |
| Biological Process | GO:0007160 | cell-matrix adhesion | 0.001854 | 0.03148 |
| Biological Process | GO:0048534 | hematopoietic or lymphoid organ development | 0.00191 | 0.032256 |
| Biological Process | GO:0032502 | developmental process | 0.00194 | 0.032601 |
| Cellular Component | GO:0044440 | endosomal part | 0.000686 | 0.032982 |
| Cellular Component | GO:0005667 | transcription factor complex | 0.000781 | 0.032982 |
| Biological Process | GO:0030801 | positive regulation of cyclic nucleotide metabolic process | 0.001989 | 0.033017 |
| Biological Process | GO:0030804 | positive regulation of cyclic nucleotide biosynthetic process | 0.001989 | 0.033017 |
| Biological Process | GO:0030808 | regulation of nucleotide biosynthetic process | 0.002006 | 0.033017 |
| Biological Process | GO:1900371 | regulation of purine nucleotide biosynthetic process | 0.002006 | 0.033017 |
| Biological Process | GO:0006810 | transport | 0.002043 | 0.033451 |
| Biological Process | GO:0012501 | programmed cell death | 0.002144 | 0.034663 |
| Biological Process | GO:0032501 | multicellular organismal process | 0.002145 | 0.034663 |
| Biological Process | GO:0018298 | protein-chromophore linkage | 0.00216 | 0.034663 |
| Biological Process | GO:0043301 | negative regulation of leukocyte degranulation | 0.00216 | 0.034663 |
| Biological Process | GO:0032611 | interleukin-1 beta production | 0.002189 | 0.034948 |
| Cellular Component | GO:0005774 | vacuolar membrane | 0.000968 | 0.035722 |
| Cellular Component | GO:0008305 | integrin complex | 0.001065 | 0.035722 |
| Cellular Component | GO:0098636 | protein complex involved in cell adhesion | 0.001387 | 0.035722 |
| Cellular Component | GO:0044437 | vacuolar part | 0.001486 | 0.035722 |
| Cellular Component | GO:0005887 | Integral component of plasma membrane | 0.001549 | 0.035722 |
| Cellular Component | GO:0016020 | Membrane | 0.001561 | 0.035722 |
| Cellular Component | GO:0044798 | Nuclear transcription factor complex | 0.001585 | 0.035722 |
| Biological Process | GO:1902105 | Regulation of leukocyte differentiation | 0.002266 | 0.035995 |
| Biological Process | GO:0045637 | Regulation of myeloid cell differentiation | 0.002368 | 0.037322 |
| Biological Process | GO:0032675 | Regulation of interleukin-6 production | 0.002373 | 0.037322 |
| Biological Process | GO:0043066 | Negative regulation of apoptotic process | 0.002407 | 0.037511 |
| Biological Process | GO:0034109 | Homotypic cell-cell adhesion | 0.002414 | 0.037511 |
| Biological Process | GO:0009628 | Response to abiotic stimulus | 0.00242 | 0.037511 |
| Biological Process | GO:0031281 | Positive regulation of cyclase activity | 0.002464 | 0.037689 |
| Biological Process | GO:0080134 | Regulation of response to stress | 0.002469 | 0.037689 |
| Biological Process | GO:0045087 | Innate immune response | 0.002478 | 0.037689 |
| Biological Process | GO:0030098 | Lymphocyte differentiation | 0.002478 | 0.037689 |
| Biological Process | GO:0045088 | Regulation of innate immune response | 0.0025 | 0.03785 |
| Biological Process | GO:0003207 | Cardiac chamber formation | 0.002582 | 0.038531 |
| Biological Process | GO:0033004 | Negative regulation of mast cell activation | 0.002582 | 0.038531 |
| Biological Process | GO:1900272 | Negative regulation of long-term synaptic potentiation | 0.002582 | 0.038531 |
| Biological Process | GO:0051349 | Positive regulation of lyase activity | 0.002636 | 0.039164 |
| Biological Process | GO:0016311 | Dephosphorylation | 0.002693 | 0.039588 |
| Biological Process | GO:0060326 | Cell chemotaxis | 0.002701 | 0.039588 |
| Biological Process | GO:0008219 | Cell death | 0.002714 | 0.039588 |
| Biological Process | GO:0016265 | Death | 0.002714 | 0.039588 |
| Biological Process | GO:0050865 | Regulation of cell activation | 0.00279 | 0.040509 |
| Biological Process | GO:0043069 | Negative regulation of programmed cell death | 0.002813 | 0.040521 |
| Biological Process | GO:0043277 | Apoptotic cell clearance | 0.002816 | 0.040521 |
| Biological Process | GO:0060548 | Negative regulation of cell death | 0.002914 | 0.041627 |
| Biological Process | GO:1903557 | Positive regulation of tumor necrosis factor superfamily cytokine production | 0.002919 | 0.041627 |
| Biological Process | GO:0016477 | Cell migration | 0.002936 | 0.041691 |
| Biological Process | GO:0071495 | Cellular response to endogenous stimulus | 0.002966 | 0.04193 |
| Biological Process | GO:0001973 | Adenosine receptor signaling pathway | 0.003038 | 0.042574 |
| Biological Process | GO:0002283 | Neutrophil activation involved in immune response | 0.003038 | 0.042574 |
| Biological Process | GO:0032635 | Interleukin-6 production | 0.003082 | 0.043005 |
| Biological Process | GO:0050764 | Regulation of phagocytosis | 0.003154 | 0.043819 |
| Biological Process | GO:0032680 | Regulation of tumor necrosis factor production | 0.00317 | 0.043842 |
| Biological Process | GO:0002429 | Immune response-activating cell surface receptor signaling pathway | 0.003199 | 0.044053 |
| Mol Function | GO:0005515 | Protein binding | 0.000297 | 0.0441 |
| Biological Process | GO:0042110 | T cell activation | 0.003384 | 0.046203 |
| Biological Process | GO:0070489 | T cell aggregation | 0.003384 | 0.046203 |
| Biological Process | GO:0030593 | Neutrophil chemotaxis | 0.003403 | 0.046267 |
| Biological Process | GO:0032640 | Tumor necrosis factor production | 0.003442 | 0.04661 |
| Biological Process | GO:0071593 | Lymphocyte aggregation | 0.003479 | 0.046726 |
| Biological Process | GO:0001820 | Serotonin secretion | 0.00353 | 0.046726 |
| Biological Process | GO:0002281 | Macrophage activation involved in immune response | 0.00353 | 0.046726 |
| Biological Process | GO:0002495 | Antigen processing and presentation of peptide antigen via MHC class II | 0.00353 | 0.046726 |
| Biological Process | GO:0035358 | Regulation of peroxisome proliferator activated receptor signaling pathway | 0.00353 | 0.046726 |
| Biological Process | GO:1903706 | Regulation of hemopoiesis | 0.003538 | 0.046726 |
| Biological Process | GO:0007165 | Signal transduction | 0.00362 | 0.047604 |
| Biological Process | GO:0002449 | Lymphocyte mediated immunity | 0.003707 | 0.048382 |
| Biological Process | GO:0045089 | Positive regulation of innate immune response | 0.003709 | 0.048382 |
| Biological Process | GO:0019538 | Protein metabolic process | 0.003785 | 0.048965 |
| Biological Process | GO:0042035 | Regulation of cytokine biosynthetic process | 0.003799 | 0.048965 |
| Biological Process | GO:0046849 | Bone remodeling | 0.003799 | 0.048965 |
| Biological Process | GO:0070486 | Leukocyte aggregation | 0.00383 | 0.049156 |
